# Isolated cell behavior drives the evolution of antibiotic resistance

**DOI:** 10.15252/msb.20145888

**Published:** 2015-07-30

**Authors:** Tatiana Artemova, Ylaine Gerardin, Carmel Dudley, Nicole M Vega, Jeff Gore

**Affiliations:** 1 Department of Physics, Massachusetts Institute of TechnologyCambridge, MA, USA; 2 Department of Systems Biology, Harvard Medical SchoolBoston, MA, USA

**Keywords:** antibiotic resistance, beta-lactamase, cooperative growth, evolution

## Abstract

Bacterial antibiotic resistance is typically quantified by the minimum inhibitory concentration (MIC), which is defined as the minimal concentration of antibiotic that inhibits bacterial growth starting from a standard cell density. However, when antibiotic resistance is mediated by degradation, the collective inactivation of antibiotic by the bacterial population can cause the measured MIC to depend strongly on the initial cell density. In cases where this inoculum effect is strong, the relationship between MIC and bacterial fitness in the antibiotic is not well defined. Here, we demonstrate that the resistance of a single, isolated cell—which we call the single-cell MIC (scMIC)—provides a superior metric for quantifying antibiotic resistance. Unlike the MIC, we find that the scMIC predicts the direction of selection and also specifies the antibiotic concentration at which selection begins to favor new mutants. Understanding the cooperative nature of bacterial growth in antibiotics is therefore essential in predicting the evolution of antibiotic resistance.

## Introduction

Predicting the evolution of antibiotic resistance in bacterial populations is a key challenge (Madigan *et al*, 2009), as the spread of antibiotic resistance has been of increasing concern worldwide (Normark & Normark, 2002). Antibiotics are used both in the clinic and for agriculture, and in addition are produced naturally by many organisms, meaning that antibiotics are present in diverse ecological environments at a wide variety of concentrations (Martínez, 2008). To predict—and possibly prevent—the spread of antibiotic resistance, we must understand the environmental conditions that select for an increase of resistance and what determines the evolutionary fitness of resistant strains. Since the term “fitness” can have different meanings depending on the context, we clarify that here fitness refers to the ability of a strain to spread in the presence of an antibiotic.

Antibiotic resistance in microbes is typically quantified by the minimum inhibitory concentration (MIC) (Andrews, 2001; Wiegand *et al*, 2008; Clinical and Laboratory Standards Institute, 2012), which is defined as the lowest concentration of antibiotic that will inhibit bacterial growth over a 20-h period in cultures starting from a standard initial cell density (Clinical and Laboratory Standards Institute, 2009). The MIC has been used as a proxy for bacterial fitness in the presence of antibiotics (Weinreich *et al*, 2006; Lee *et al*, 2010; Tan *et al*, 2011), and in addition is sometimes thought to indicate the minimal antibiotic concentration at which there is selection for increased resistance (Yeh *et al*, 2009; Hermsen *et al*, 2012). Thus, the MIC plays an important role in our understanding of the evolution of antibiotic resistance in bacteria.

**Figure 1:**
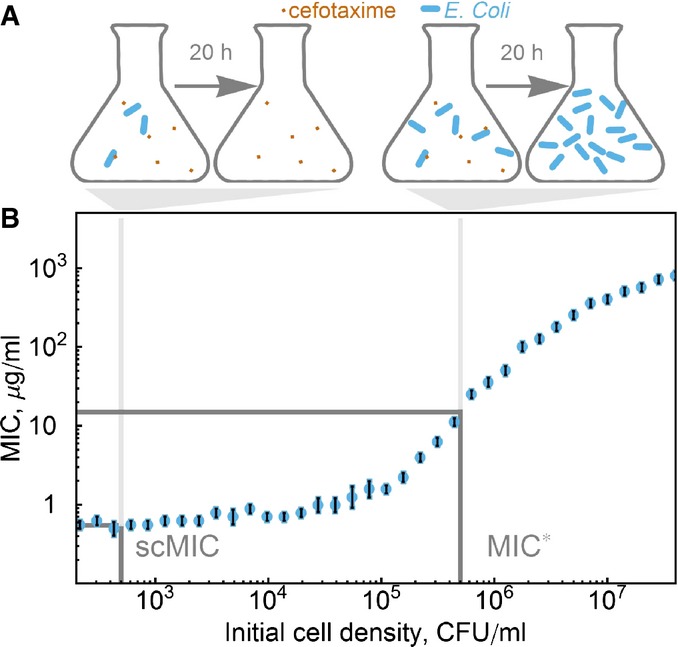
The measured minimum inhibitory concentration (MIC) levels off in the limit of small densities, asymptotically approaching the single-cell MIC (scMIC) Design of the inoculum effect experiment. The initial cell density determines whether in 20 h, the population survives at a given antibiotic concentration. On the left, the cell density is not enough to produce the necessary amount of enzyme to break down the antibiotic. Therefore, in 20 h all cells are dead. On the right, the cell density is high enough to produce enough enzymes, and therefore, in 20 h the population survives the treatment and no antibiotic is left in the media.

However, while the MIC has been used as a single value proxy for fitness, its relationship to evolutionary fitness is often complicated. For β-lactam antibiotics, the oldest and most widely used class of antibiotics (Bonomo & Tolmasky, 2007), the MIC is frequently subject to the “inoculum effect”: Its measured value is strongly dependent upon the starting cell density of the culture (Brook, 1989; Meredith *et al*, 2015) (Fig1). This occurs because resistance to β-lactams is often achieved via hydrolytic inactivation of the antibiotic by resistant cells, which can benefit the entire bacterial population by causing overall depletion of antibiotic (Dugatkin *et al*, 2005; Clark *et al*, 2009). β-lactams are bactericidal, and therefore, any bacterial population that survives the treatment will often go through a phase of cell death. The dynamics of these populations can be complex (Yurtsev *et al*, 2013), and since the MIC is sensitive to the initial cell density, the relevance of a high-density MIC measurement to the evolutionary fitness of individual bacteria is unclear (Goldstein *et al*, 1991).

We define the scMIC as the measured MIC at low starting cell densities. The measured MIC of TEM-20 (reference) strain varies by three orders of magnitude depending upon the starting cell density and asymptotically approaches a limit at low cell densities. The gray bars correspond to the initial cell densities for MIC* and scMIC. The error bars are the maximum of a discretization error and the standard error of the mean of three measurements. Source data are available online for this figure.

In this paper, we demonstrate that the MIC is in many ways a flawed metric for quantifying the fitness of antibiotic-resistant bacteria in β-lactam antibiotics because the MIC depends upon the cooperative growth dynamics between cells. We find that measuring the direct benefit conferred by resistance for a single, isolated cell is a more robust, meaningful, and useful way to quantify the fitness of a resistant bacterial strain. This single-cell resistance is simply the MIC measured in the limit of low initial cell density, which we call the single-cell MIC (scMIC). This quantity predicts both the direction of selection and the approximate antibiotic concentration at which there is selection for increased resistance. Importantly, these two key predictive properties of the scMIC are independent of the density of the bacterial culture in which selection occurs, and thus, the scMIC can provide valuable guidance for researchers and clinicians studying the evolution of antibiotic resistance.

## Results

### Measurement of single-cell MIC

In this study, we use the β-lactam antibiotic cefotaxime and β-lactam-resistant *E. coli* strains (Weinreich *et al*, 2006) to quantify the evolutionary predictive power of the MIC. Each resistant strain expresses a plasmid-encoded TEM β-lactamase enzyme, which can hydrolytically inactivate a wide range of targets (Bush *et al*, 1995; Jacoby, 2006), including the third-generation cephalosporin cefotaxime (Stemmer, 1994; Hall, 2002).

Throughout the remainder of this paper, we use the abbreviation “MIC” to describe the lowest concentration of antibiotic that inhibits growth of a culture over 20 h; this MIC is a function of the initial cell density. We will denote the specific MIC value for the standard initial cell density (standard density is 5 × 10^5^ cells/ml) as the MIC*.

Consistent with previous measurements (Brown *et al*, 1981), we observed the inoculum effect in β-lactam-resistant *E. coli* TEM strains: The MIC often depends strongly on the initial cell density. In particular, the MIC increases dramatically at high cell densities but plateaus at low initial cell densities. For example, the MIC for *E. coli* expressing β-lactamase TEM-20 varied by three orders of magnitude depending upon the initial cell density. As the cell density decreased, the measured MIC asymptotically approached a limit, which corresponds to the level of resistance of a single, isolated cell: the scMIC (Fig1B). Interestingly, this is also the lowest antibiotic concentration that results in cell death at a wide range of cell densities (Supplementary Fig S1). Based on these results, we standardized our measurements of the scMIC by using an initial cell density of 500 cells/ml, a thousand times smaller than the standard MIC* initial cell density.

As a metric of the level of antibiotic resistance, the scMIC has several attractive qualities. First, the scMIC can be measured in the same experimental setup as the MIC*, with the only change being a decrease in the initial cell density. Moreover, because the MIC curve plateaus at low cell density, where the scMIC is measured, scMIC measurements are also more robust against experimental errors in the initial cell density. This asymptotic limit also makes it possible to measure scMIC without diluting to the limit of single cells, thus avoiding stochastic effects associated with very small starting cell numbers.

### scMIC is the MIC of a single cell

**Figure 2:**
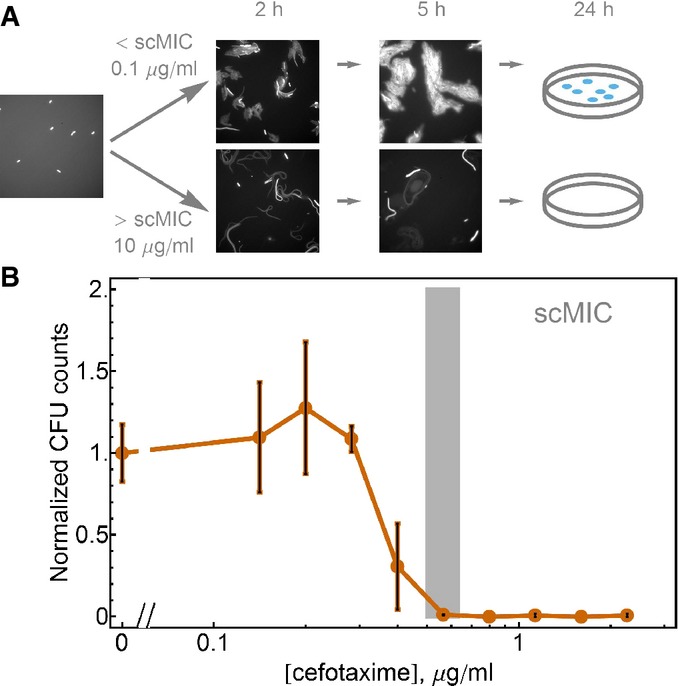
scMIC is the MIC of a single cell The diagram of the time evolution of cell density on the surface of the agar media at two antibiotic concentrations: below (the top row of images) and above (the bottom row of images) the scMIC of the imaged strain (reference strain). We initially pipette diluted saturated culture on the surface of the agar (0.4%) and take an image on which we can see distinct single cells scattered on the surface. While at antibiotic concentration below the scMIC, an exponential growth of cells is happening during the first 5 h, at the antibiotic concentration above the scMIC, cells undergo filamentation and do not form colonies in 1 day.

To demonstrate that the scMIC truly measures the MIC of a single cell (not only in the limit of diluting to single-cell density), we monitored the behavior of a single cell on agar with various antibiotic concentrations (Fig2A). Initially, single cells were scattered at low density on the agar surface. After 2 h, cells exposed to low antibiotic concentrations formed microcolonies, while cells exposed to antibiotic concentrations higher than the scMIC grew as filaments. Filament formation has previously been observed in bacteria exposed to antibiotics (Chung *et al*, 2009; Yao *et al*, 2012); filamentation leads to cell death and the failure to form macroscopic colonies on agar.

The scMIC can also be estimated by plating cells on agar and counting colony growth after overnight incubation. Saturated cultures of the reference strain TEM-20 were evenly spread on agar plates with various cefotaxime concentrations. The colony-forming units (CFU) were evaluated for two independent cultures and normalized by the CFU obtained without antibiotics. The error bars are the maximum of the two Poissonian errors for zero antibiotic concentration and the standard error of the mean for all non-zero antibiotic points. Source data are available online for this figure.

We next compared the antibiotic concentration required to prevent colony formation after overnight growth with the scMIC measured in liquid culture. These quantities should be equivalent: each colony observed on an agar plate develops from a single cell that was able to reproduce in a given antibiotic environment. Encouragingly, we found that the single-cell resistance measured on agar is within a factor of two of the scMIC obtained by the liquid dilution method (Fig2B), with both quantities being at least an order of magnitude smaller than the MIC*. The fact that very different experimental approaches yield similar quantities gives us confidence that both methods are indeed quantifying the resistance of a single, isolated cell.

### Selection starts at the scMIC even if cell density is high

**Figure 3:**
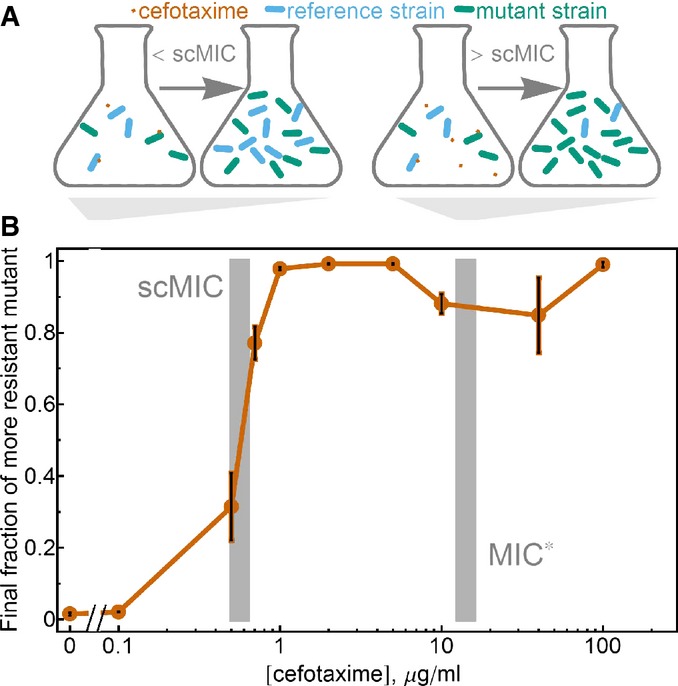
Selection starts at the scMIC even if the cell density is high Design of the competition experiment. The mixtures of reference and mutant strain were competed overnight in media with different cefotaxime concentrations. When the initial cefotaxime concentration is below the scMIC of the reference strain, the mutant strain does not have a selective advantage and its fraction remains unchanged after overnight incubation. When the initial antibiotic concentration is above the scMIC of the reference strain, the mutant strain has higher fitness and its fraction in the population increases after overnight incubation.

Given that the scMIC of a strain is often significantly lower than its MIC*, an important question is what antibiotic concentrations will lead to selection of one strain over another when two strains are in competition, that is, which conditions promote the evolution of increased resistance. Antibiotic concentrations below the scMIC of both competing strains are not expected to be strongly selective. A mutant with a higher scMIC value than the background population gains a relative advantage when the antibiotic concentration is at least the scMIC of the background population. At that concentration, the background population will begin to die, but the mutant will not. As a result, below the scMIC of the reference strain, the fractional composition of the bacterial population will not change overnight, while if the antibiotic concentration in the environment is above the scMIC of the reference strain, the mutant will rapidly increase in fraction (Fig3A). Importantly, this prediction should hold true even if the population density is high and the overall MIC of the entire population is therefore higher than the scMIC of either of the two strains.

In competition of the reference and mutant strain, selection for the more resistant mutant begins at antibiotic concentrations near the scMIC, not the MIC*, of the reference strain. Competition experiments were performed with the YFP and CFP plasmids swapped between the reference and mutant strains; no difference in final fraction of the mutant between these two types of labeling was observed. The error bars are the standard error of the mean (*n *=* *8–9 expect for last two data points where *n *=* *6 and 2). The gray bars correspond to the MIC* and scMIC values of the reference strain ± s.e.m. Source data are available online for this figure.

We tested this prediction experimentally by directly competing what we call the reference strain (TEM-20, scMIC 0.65 μg/ml) with its high scMIC mutant (8 μg/ml, mutation E104K). In this experiment, the two strains were labeled with plasmids expressing either yellow or cyan fluorescent proteins, thus allowing us to measure fractions by flow cytometry; labels were swapped in replicates of these experiments (Supplementary Fig S2). Consistent with the argument in the previous paragraph, selection favoring the E104K mutant began when the antibiotic concentration approached the scMIC of the reference strain (Fig3B). Note that the cell density in this competition experiment was high enough so that the MIC measured at this cell density is higher than the scMIC of either strain; although scMIC might be measured at low cell density, it nonetheless provides guidance about selection at higher cell densities where collective inactivation of the antibiotic is significant. To demonstrate the generality of our claim that selection starts in the vicinity of the scMIC of the background population, we confirmed that this was also the case for competition between the reference strain and another mutant (A42G mutation, scMIC 1.6 μg/ml) (Supplementary Fig S3). Selection for a higher fitness mutant therefore begins when the antibiotic concentration reaches the scMIC of the background population, which is often an order of magnitude lower than the MIC* of the population.

**Figure 4:**
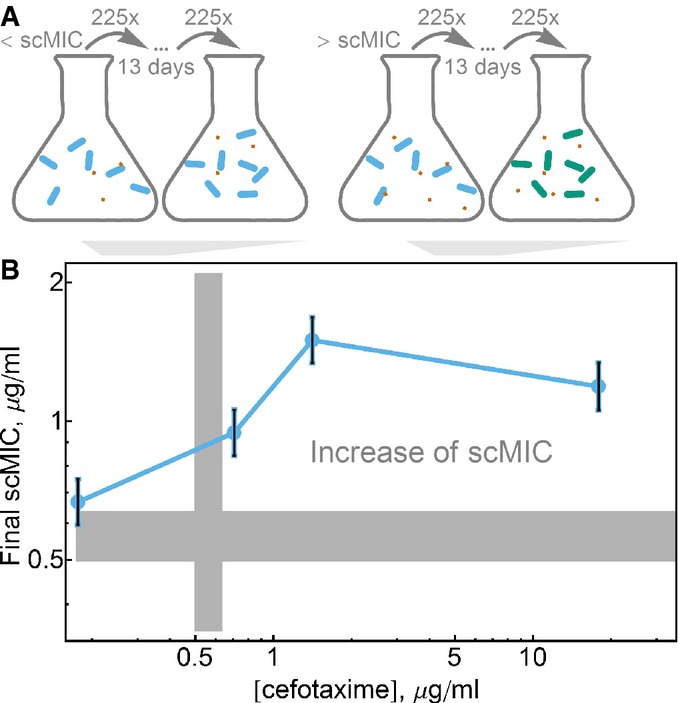
Higher levels of resistance evolve at antibiotic concentrations above scMIC Design of the laboratory evolution experiment. Identical clonal populations of cells were evolved over 13 days with daily dilution by 225 in media with various antibiotic concentrations. The increase of the scMIC of the population at the end of the experiment was observed in the media where antibiotic concentrations were above scMIC of the initial population.

Following the logic in the previous section, the scMIC of a population naturally evolving in the presence of antibiotics should increase over time, as long as the antibiotic concentration is high enough to exert a selective pressure. The scMIC is also expected to predict the antibiotic concentration where *de novo* mutants with higher scMIC can arise. To test this prediction, we performed laboratory evolution experiments at multiple antibiotic concentrations both above and below the scMIC of the starting strain (Fig4). In these experiments, we evolved six replicates of our reference strain *E. coli* at four different cefotaxime concentrations for ∼100 generations (daily dilutions by 225× for 13 days, Fig4A). As expected from our competition experiments, the cultures that were evolved at antibiotic concentrations lower than the scMIC of the starting populations displayed no increase in the scMIC. In contrast, cultures that were evolved at antibiotic concentrations higher than or equal to the ancestral scMIC displayed a significant increase in resistance as measured by the scMIC (Fig4B). Note that the effective cell densities at which the populations were evolved are much higher than the cell density at which the scMIC is measured. To confirm the generality of these results, we performed laboratory evolution on two other strains carrying different versions of β-lactamase, and once again found that only the populations evolved at antibiotic concentrations close to or larger than the ancestral scMIC evolved an increase in scMIC (Supplementary Fig S4).

Laboratory evolution experiments of the reference strain confirm that increase of resistance evolves in antibiotic concentrations equal to and larger than the scMIC. Plotted is the scMIC measured after 13 days versus the concentration of cefotaxime the strains were evolved in. The error bars are the standard errors of the mean of six independently evolved populations. The gray bars correspond to the initial scMIC values ± s.e.m. Source data are available online for this figure.

### *In vivo* relevance of scMIC

A reasonable concern is that the competition outcome and dynamics between bacterial strains could be qualitatively different during growth inside a host. To explore this, we used the nematode worm *Caenorhabditis elegans*, a widely used model host that can be infected and killed by a variety of human pathogens (Moy *et al*, 2006; Paulander *et al*, 2007; Ewbank & Zugasti, 2011). *Caenorhabditis elegans* has been proposed as a model system for tests of antimicrobial efficacy, with improved pharmacokinetics as compared with traditional *in vitro* analysis (Moy *et al*, 2006), and may therefore be useful for assessing the generality of antibiotic treatment-driven dynamics.

**Figure 5:**
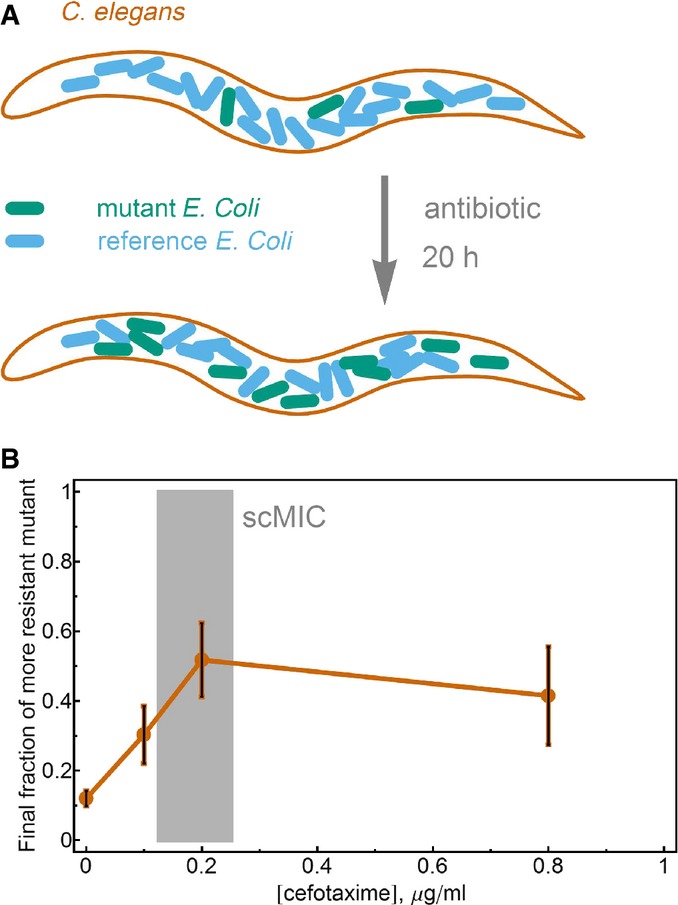
The scMIC predicts selection in an animal infection model Schematic representation of the *in vivo* experiment. *C. elegans* were colonized with a mixture of the reference strain (90%) and the more resistant mutant TEM-52 (10%) and were treated with antibiotic for 20 h before the final fraction of TEM-52 was measured.

Briefly, synchronized adult *C. elegans* were fed on a mixture of 90% reference strain and 10% mutant strain to establish a mixed gut community and were then incubated in worm media containing varying cefotaxime concentrations (Fig5A). After 20 h of antibiotic treatment, we mechanically disrupted the worms to release gut-associated bacteria and measured the strain composition of *E. coli* by plating. These experiments were performed at low temperatures (23°C) to prevent heat shock and death of the worms.

Selection for increased resistance begins at antibiotic concentrations near the scMIC, as in the *in vitro* measurements. The error bars are the standard error of the mean of three measurements. The discrepancy between the *in vivo* scMIC and the *in vitro* measurements is likely due to differences in respective environmental conditions, such as nutrient availability and temperature. Source data are available online for this figure.

Consistent with our *in vitro* competition experiments described above, we found that selection for the more resistant mutant (TEM-52) starts at the scMIC value of the less resistant background allele (Fig5B, note that the scMIC of the reference strain, TEM-20, is different in these conditions (Björkman *et al*, 2000)). Our key observation that selection for increased resistance occurs at the vicinity of the scMIC rather than the MIC* is therefore valid both in direct liquid culture experiments and in the very different environment of a simple animal host.

### Model

**Figure 6:**
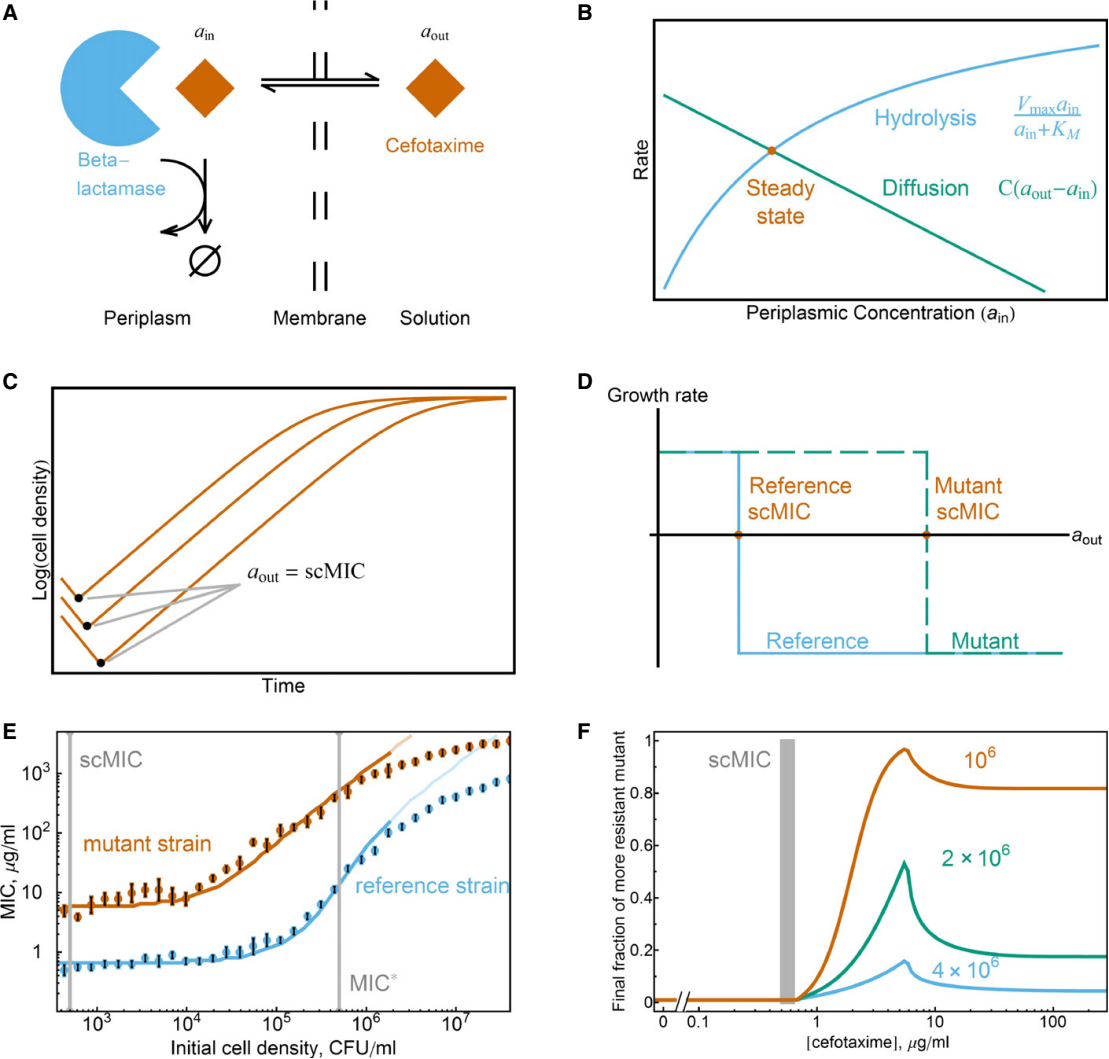
A simple model captures predictive power of the scMIC Cefotaxime diffuses into the periplasmic space of the cell, where the enzyme β-lactamase hydrolyzes cefotaxime. *a*_in_ and *a*_out_ correspond to the cefotaxime concentrations in the periplasmic space and outside of the cell, respectively.

We developed a simple model to better understand the inoculum effect and the evolutionary meaning of the scMIC and MIC*. In this model, antibiotic diffuses into the periplasmic space of a bacterial cell to inhibit cell wall synthesis. Resistant bacteria secrete the β-lactamase enzyme into the periplasmic space, where it inactivates the antibiotic (Walsh, 2000) (Fig6A). At steady state, the flux of antibiotic into the periplasmic space equals the rate that the enzyme inactivates the antibiotic. The resulting active antibiotic concentration in the periplasmic space is therefore lower than the concentration outside of the cell (Zimmermann & Rosselet, 1977) (Fig6B). We assume that the division rate of the cell is a function only of the periplasmic antibiotic concentration, which depends upon both the extracellular antibiotic concentration and the enzyme kinetics.

At steady state, the diffusion rate of cefotaxime into the cell equals the Michaelis–Menten hydrolysis rate of cefotaxime within the cell. The corresponding cefotaxime concentration inside the periplasmic space is therefore smaller than the concentration outside the cell. *C* is a permeability parameter; *V*_max_ and *K*_M_ characterize the hydrolytic activity of the enzyme.

Bacterial growth curves with the same initial antibiotic concentrations but different starting densities. The cells die until the external concentration of cefotaxime reaches the scMIC of the strain.

The growth/death rate is a step function of the external cefotaxime concentration, as this determines the periplasmic concentration. Strains with different versions of TEM enzyme will have different scMIC values, which is the external antibiotic concentration at which the growth rate becomes negative (death).

The fits of the inoculum effect curves of the reference and mutant strains (dark regions correspond to the fitting interval). The error bars are the maximum of a discretization error and the standard error of the mean of three measurements.

The model prediction for competition experiments, with a 1% initial fraction of the mutant. At the scMIC of the reference strain, the final fraction of the mutant strain starts to increase, indicating that selection for the more resistant mutant starts near the scMIC. Different colors correspond to different initial cell densities (labeled in CFU/ml). The gray bar corresponds to the scMIC of reference strain. For the model, parameter values are provided in the Supplementary Information. Source data are available online for this figure.

We experimentally found that for our TEM strains in cefotaxime, this growth rate function can be approximated as a step function: Cells divide at a normal rate until the antibiotic concentration in the periplasmic space is above some value *a*_crit_, at which point the cells die at a rate ∼2 per h (Fig6C and D; Supplementary Figs S1 and S5). Increased resistance in our experiments is conferred with no cost because it is acquired by one or a few point mutations in β-lactamase. The benefit of the higher resistance (and therefore lower periplasmic concentrations for a given concentration outside the cell) is realized only when the antibiotic concentration is high enough so that the reference strain dies and low enough so that the mutant strain still grows. Outside of this antibiotic concentration range, there is no benefit of higher resistance.

In this model, the scMIC of a strain is the external concentration of antibiotic that gives rise to a periplasmic concentration of *a*_crit_. A non-resistant strain that cannot inactivate antibiotics has a periplasmic concentration of antibiotic approximately equal to the external concentration, suggesting that *a*_crit_ is simply the scMIC of a sensitive strain. Therefore,

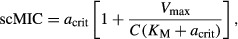
where *C* is the permeability of the membrane, and *V*_max_ and *K*_M_ are the Michaelis–Menten parameters of the enzyme (the maximum reaction rate of the enzyme and the substrate concentration at which the reaction rate is half of *V*_max_, respectively). Thus, within this model, a mutant strain with a more efficient enzyme (higher *V*_max_ and/or lower *K*_M_) will have a higher scMIC. This equation has been proposed to quantify MIC* (Nikaido & Normark, 1987), but this is correct only when the inoculum effect is weak and scMIC is approximately equal to MIC*.

We assume that growth above the scMIC results from a transient death phase in which β-lactamase is released into the media, contributing to the collective inactivation of the antibiotic. This simple model correctly predicts the relationship of the MIC to the initial cell density for both the reference and mutant strains (Fig6E). For each strain, there are two free parameters that describe the efficiency of the particular version of the enzyme in hydrolyzing cefotaxime: the Michaelis–Menten parameters *V*_max_ and *K*_M_. Our model also provides insight into the upper bound for the cell density that should be used in the definition of scMIC. For the strains that we use, the density should be < 10^4^ cells per ml (see Supplementary Information for the reasoning), which our proposed definition of scMIC indeed satisfies.

In our model, the inoculum effect curve can be derived analytically in the limit of low and high cell densities. These two regimes are determined by how MIC compares to *K*_M_. If MIC is smaller than *K*_M,_ then the hydrolysis rate increases proportionally to the antibiotic concentration, and as a result, the measured MIC is an exponential function of the initial cell density:

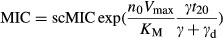
where *n*_0_ is the initial cell density, *γ* is the growth rate, *γ*_d_ is the death rate, and *t*_20_ is the time before evaluation of the MIC (usually 20 h). At higher antibiotic concentrations, the hydrolysis rate becomes independent of the antibiotic concentration, and the model predicts that MIC increases linearly with the initial cell density. However, the experimentally measured MIC grows slower than linearly with the initial cell density. This phenomenon could be explained by the fact that in this regime, the population spends a significant amount of time in the death phase and degradation of the released enzyme could become significant (Supplementary Information).

Our model agrees with the experimental finding that independent of initial cell density, selection favoring the competitor with the higher scMIC will begin when the antibiotic concentration approaches the scMIC of the less resistant strain (Fig6F). In our experiments, selection starts at even somewhat lower antibiotic concentrations than predicted by our model (Fig3B), likely because this minimalist model assumes that the antibiotic has no effect until the cell begins to die (see Fig6D); a gradual decrease in the growth rate with antibiotic concentration would result in selection at concentrations below the scMIC. In either case, selection may occur at antibiotic concentrations that are orders of magnitude lower than the MIC*.

Our model also predicts that the strength of selection for increased scMIC will depend non-monotonically on the antibiotic concentration, leading to the counter-intuitive effect whereby adding additional antibiotic decreases the ability of a mutant with higher scMIC to spread against the background population (Fig6F). This surprising prediction was also validated in both our *in vitro* and *in vivo* experiments (Figs3 and 4). The strength of selection decreases above the scMIC of the winning strain because the released enzyme from the dying bacteria (Sykes & Matthew, 1976) hydrolyses antibiotic faster than the rate of antibiotic hydrolysis within a cell. This effect can be understood within a framework of altruistic death, either deterministic (Tanouchi *et al*, 2012) or stochastic (Ackermann *et al*, 2008), in which the death is favorable for the population if the benefit from the released public goods is strong enough.

Finally, the model also successfully predicts that lower initial cell densities will experience stronger selection (Fig6F; Supplementary Fig S6). This is because lower initial cell densities will take longer to inactivate the antibiotic, thus extending the window for selection during which the less resistant strain experiences cell death. On the other hand, the antibiotic concentration at which selection starts does not depend strongly on the cell density. This makes sense since the periplasmic antibiotic concentration at the beginning of the experiment is independent of the cell density. The cell density does, however, alter the temporal dynamics of the antibiotic concentration over the course of the day, thus modifying the strength of selection favoring the strain with higher scMIC. Although this simple model works well at low to moderate antibiotic concentrations, it does not explain the behavior of the inoculum effect and selection curves at high antibiotic concentrations. To account for both discrepancies, we could allow for degradation of the β-lactamase enzyme in the model (Supplementary Fig S7).

### MIC*-scMIC relationship

In this system, cooperative resistance and the inoculum effect occur due to enzymatic inactivation of antibiotics. The population’s collective capacity to inactivate the antibiotic is expected to increase with both cell density and the efficiency of the resistant enzyme. We therefore hypothesized that strains carrying a highly efficient enzyme (and therefore showing high scMIC) would also have a large difference between the scMIC and the high-density MIC*.

**Figure 7:**
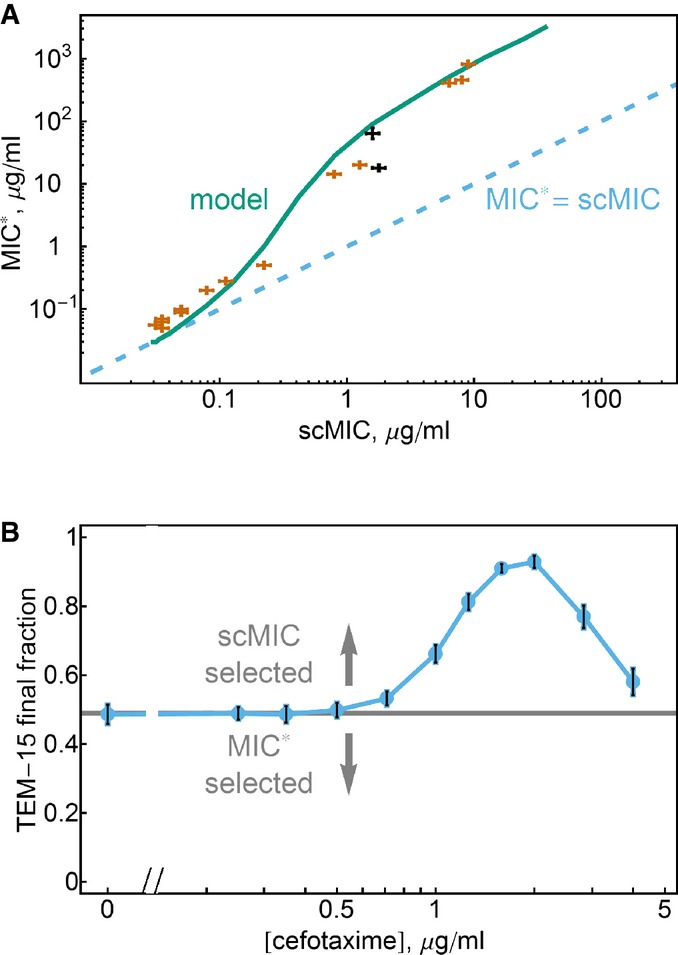
While scMIC–MIC relation can be complex, the scMIC always determines the direction of selection MIC* and scMIC typically increase together. Our model accurately predicts the general relationship between MIC* and scMIC. Plotted is the MIC* and scMIC values for 16 different TEM mutants. Varying only *V*_max_ in our model (teal) explains the experimental trend (*K*_M_ = 10 μg/ml). MIC* = scMIC line (dashed blue) shows that MIC* and scMIC are similar for strains with low resistance, whereas MIC* is more than two orders of magnitude larger than scMIC for strains with high resistance. TEM-15 and the A42G mutant of TEM-20 are black. Error bars are the maximum of the standard error of the mean of three measurements and a discretization error associated with the microdilution method (see Materials and Methods).

To characterize this relationship, we measured the MIC* and scMIC in cefotaxime for 16 *E. coli* strains with different versions of the TEM β-lactamase enzyme (Fig7A) (Weinreich *et al*, 2006). We found that the inoculum effect is strong for all the highly resistant strains, with the MIC* often being two orders of magnitude higher than the scMIC. However, at low levels of resistance, the MIC* and scMIC values are nearly the same. Our model is able to explain this relationship between the MIC* and the scMIC by assuming that all of the strains are equivalent except for variation in the *V*_max_ of the β-lactamase enzyme (though *in vitro* measurements indicate that both *V*_max_ and *K*_M_ are sensitive to mutations in the enzyme (Philippon *et al*, 1989; Wang *et al*, 2002)).

Selection favors an increase of scMIC not MIC*. The competition experiment of TEM-15 and the A42G mutant of TEM-20 (initial fraction plotted as horizontal line, initial cell density 5 × 10^5^ cells/ml). TEM-15 has a somewhat higher scMIC (1.78 μg/ml vs. 1.59 μg/ml), while the A42G mutant of TEM-20 has a much higher MIC* (64 μg/ml vs. 18 μg/ml). For cefotaxime concentrations above the scMIC of the A42G mutant of TEM-20, the TEM-15 strain is selected for, indicating that selection maximizes the scMIC rather than the MIC*. Error bars are the standard errors of the mean of four independent populations. Source data are available online for this figure.

Although in general the MIC* increases together with the scMIC, we found some exceptions (Fig7A). For example, the A42G mutant of TEM-20 has an MIC* that is almost four times larger than that of TEM-15 (64 μg/ml vs. 18 μg/ml; pair drawn in black in Fig7A). Nevertheless, our measured scMIC for TEM-15 is if anything somewhat higher (1.59 μg/ml with 68% confidence interval (1.41; 1.78) vs. 1.78 μg/ml with 68% confidence interval (1.59; 2)). The MIC* and scMIC can have different orderings because the MIC* reflects the cooperative hydrolysis of the antibiotic at high cell density (and often scales with *V*_max_ of the enzyme), whereas scMIC reflects the “selfish” hydrolysis in the periplasmic space (and scales as the ratio of *V*_max_*/K*_M_ for large enough *K*_M_). This distinction may be relevant for other resistance mechanisms governed by enzymatic inactivation of a drug. Enzymatic inactivation has been an observed mechanism of resistance against several classes of antibiotics, including β-lactams, aminoglycosides (Shaw *et al*, 1993) and macrolides (Leclercq, 2002), suggesting that these ideas may have broad relevance in the study of antibiotic resistance.

Given that both the MIC* and the scMIC are intended to measure the level of resistance, this prompts the question: which strain is favored in the presence of the antibiotic? More generally, does selection favor an increase in MIC*, as is generally assumed, or does selection instead favor an increase in scMIC as we argue here? We competed the two strains and found that the antibiotic selects for TEM-15 (Fig7B), suggesting that selection does indeed maximize the scMIC rather than the MIC*. To confirm that this conclusion is not specific to this particular pair of strains, we repeated the competition experiments with another pair exhibiting a reversal between scMIC and MIC* and obtained similar results (Supplementary Fig S8). Finally, we analyzed the evolved lines in Fig1C and found several cases in which laboratory evolution led to an increase in scMIC but no discernible increase in the MIC* (Supplementary Fig S9); a decrease in MIC* has also been observed in laboratory fungal evolution (Cowen *et al*, 2001)). Taken together, these results argue strongly that selection acts on the scMIC rather than the MIC*, since the scMIC is the quantity that directly measures the fitness of an individual cell.

## Discussion

Understanding the role of collective resistance in bacterial antibiotic response is essential in predicting the evolution of antibiotic resistance. When mutants arise in an antibiotic-resistant population, the MIC* is often thought to indicate both the direction of selection and the approximate antibiotic concentrations that will lead to strong selection for increased resistance. We have found here that for β-lactams, the MIC* can fail in both of these tasks and found instead that the scMIC—the resistance of a single, isolated cell—accurately predicts the evolutionary behavior of bacterial populations exposed to an antibiotic.

While the scMIC is a better way of predicting evolution than the MIC*, the MIC* still contains important information that could be used for purposes other than predicting evolution. For instance, the MIC* captures the population-level resistance due to effects such as the collective inactivation of a drug. This population-level resistance is useful for determining proper antibiotic dosage and regimen because the entire population of many cells needs to be killed, and therefore, the cooperative part of resistance cannot be ignored. It is important to stress that predicting evolution and estimating the antibiotic concentration required to kill a population of a given size are very different questions; while the former requires understanding the costs and benefits to a single cell, the latter requires quantification of the population-level resistance.

While selection typically favors individuals with higher fitness, under some circumstances selection can favor genotypes with higher group fitness even at the expense of individual fitness. These “cooperative” genotypes can be favored as the result of population structure that facilitates group/kin selection (Nowak, 2006; Damore & Gore, 2012). One example of such structure is spatial organization that leads to competition between subpopulations with different genotypes. In this case, selection will act partially on these subpopulations, and it may possible to select for a strain with higher MIC* at the expense of a decrease in scMIC.

The results of this paper hold if the following assumptions about the antibiotic resistance can be made: (i) There is no cost in being more resistant, (ii) the resistance mechanism is cooperative, and (iii) there is no gradual dependence of the growth rate on the antibiotic concentration: For any concentration below, the scMIC growth is exponential with a rate that does not depend on the antibiotic concentration; above the scMIC, death is exponential with a rate that is again independent of the antibiotic concentration.

The first assumption is applicable here because we study the evolution of a protein and consider very few mutations that change its enzymatic properties. This assumption generally can be made whenever small improvements of already existing adaptations drive the evolution, as compared, for example, to the acquisition of a plasmid carrying an antibiotic resistance gene.

The second assumption states that the effect of the global decrease of antibiotic due to hydrolysis is significant when compared to the effect of the local decrease of periplasmic antibiotic concentration due to partial localization of the enzyme. Broadly, whenever the global effects of antibiotic breakdown are strong enough as compared to local, this assumption is valid. Although our experiments have focused on the β-lactam cefotaxime, it is possible that similar phenomena may be observed for other drugs that show an inoculum effect. The inoculum effect can often be caused by enzymatic degradation of antibiotics, and plasmid-borne antibiotic-degrading enzymes are widespread among bacteria in natural environments (Bennett, 2008). Even when enzymatic inactivation does not occur, the inoculum effect can be generated by antibiotic titration, as has been observed for ribosome-inhibiting antibiotics (Tan *et al*, 2012). The distinction between the scMIC and the MIC* may therefore be relevant across many classes of antibiotics.

The third assumption is that the transition from growth to death is sudden as a function of the antibiotic concentration (Fig6D), which is a reasonable approximation for most β-lactams and for a variety of other antibiotics (Wiuff *et al*, 2005; Johnson & Levin, 2013). While in reality this transition is always somewhat gradual, the relevant criterion is how the division/death time scale compares to the time it takes for the antibiotic concentration to fall from the region in which the cells are strongly affected to the region in which they are no longer significantly inhibited. If it takes the cell less than a division/death time, then the transition is sudden.

Other work has suggested a distinct mechanism by which sub-MIC* concentrations of antibiotic may select for increased resistance. For example, a recent study demonstrated that sub-MIC* levels of tetracycline, aminoglycosides, and fluoroquinolone antibiotics can select for cells carrying an antibiotic resistance plasmid (Gullberg *et al*, 2011). The resistance mechanism in this previous study was not cooperative, and inoculum effects were not observed; selection occurred when growth inhibition of sensitive strains at sub-MIC* antibiotic concentrations was greater than the growth cost associated with the plasmid conferring resistance, a point designated by the authors as the minimal selective concentration (MSC). In the study be Gullberg *et al*, therefore, very low concentrations of antibiotic have a modest but potentially significant effect on bacterial growth. In this situation, it is possible to get selection for antibiotic resistance at sub-MIC concentrations of antibiotic, even in the absence of collective inactivation of the antibiotic (in which case the scMIC is equal to the traditional MIC). Collective antibiotic degradation is therefore not the only mechanism for sub-MIC selection for antibiotic resistance.

It is important to recognize that antibiotic degradation need not produce a strong inoculum effect. When enzymatic degradation is slow, modeling and experimental results indicate that the scMIC and MIC* will be small and their values will be similar (Fig7A). In this case, we still expect selection to be non-monotonic with antibiotic concentration as observed here, where the strongest selection for a more resistant mutant occurs at drug concentrations that inhibit the background strain but allow the mutant to grow (Fig3C; Supplementary Figs S3 and S6). In fact, non-monotonic strength of selection has been observed as a function of cefotaxime concentration in *E. coli* expressing weak alleles of TEM β-lactamase (Negri *et al*, 2000) with no distinction between scMIC and MIC*.

Unlike the MIC*, the scMIC can be measured in a shorter time frame than 20 h (Fig2A). The time it takes to determine the resistance level of bacteria is crucial for patient survival (Soong & Soni, 2012), and several methods have been suggested to quantify the level of resistance within a few hours using microfluidics (Choi *et al*, 2013; Mohan *et al*, 2013). Even without microfluidics, simple microscopy of microcolony growth after a few hours can be a rapid diagnostic to determine whether a bacterial strain is resistant (Chadwick, 1966). Since these methods can determine the antibiotic concentration when the growth of a single cell is significantly inhibited, they can be used for scMIC determination. They cannot, however, be used for MIC* determination since the experiments do not probe whether a larger population of cells would have survived the antibiotic treatment after a longer period of exposure.

The resistance of the entire population—the MIC*—incorporates the cooperative nature of bacterial growth, and generally differs from the resistance of a single cell, quantified by the scMIC. Put most simply, selection acts on individuals and favors genotypes that perform better as individuals, and as such, the single-cell MIC is the proper metric for predicting which mutations will be favored by selection.

## Materials and Methods

### Strains

TEM strains were obtained from Weinreich *et al* (2006). All strains were *E. coli* DH5α transformed with pBR322 plasmids carrying different alleles of TEM-1. These alleles represented all possible combinations of the presence or absence of A42G, E104K, M182T, and G238S mutations in the β-lactamase gene.

### MIC/scMIC

For MIC/scMIC measurements, the strains were cultured at 37°C in 5 ml LB with 50 μg/ml piperacillin (for plasmid selection) for 18–20 h with 300 rpm shaking in 50-ml falcon tubes. Cultures were then diluted to the initial optical densities and grown in serial dilutions of cefotaxime in 96-well plates at 37°C for 20 h with 500 rpm shaking. Minimum inhibitory concentration was determined by the lowest concentration that prevented bacterial growth (OD < 0.3). All measurements were done in triplicate.

### Competition experiments

For competition experiments, we transformed TEM strains with plasmids constitutively expressing either CFP or YFP (plasmids pZS25O1 + 11-Cerluean and pZS25O1 + 11-YFP). Two cultures of different colors were grown from single colonies for 18–20 h in 50 μg/ml of piperacillin and 50 μg/ml of kanamycin for plasmid selection. These cultures were then mixed and grown for another 20–22 h in 50 μg/ml of piperacillin and 50 μg/ml of kanamycin to synchronize the growth phases of the two strains. The purpose of synchronization was to eliminate any experimental variability and experimental effects due to the difference in the growth phases of the two cultures in the beginning of the experiment (in particular, synchronization of the lag time is important for reproducibility). The synchronized mixed culture was diluted to multiple initial cell densities and exposed to various cefotaxime concentrations on 96-well plates. After 25 h of growth at 37°C with shaking, the cultures were diluted in PBS 1:900 and measured at the flow cytometer. For the competition of TEM-15 and A42G mutant of TEM-20, the second day of growth of the two strains together before the addition of cefotaxime was done with no piperacillin present. The reason for that is that piperacillin scMIC of TEM-15 is smaller than 50 μg/ml and the prepared initial fraction shifted significantly over the course of 20–22 h growth. We confirmed that this did not happen with the other strains that we used for competition experiments.

### Competition experiments in a *C. elegans* model

Synchronized cultures of adult *C. elegans* were produced according to standard protocols (Stiernagle, 2006). Unless otherwise specified, all experiments were performed at 23°C; liquid culture experiments were performed with shaking at 300 rpm. Asynchronous cultures of the temperature-sensitive sterile mutant *C. elegans* AU37 were grown at permissive temperatures (16°C) on NGM agar plates with *E. coli* OP50 as a food source; recently starved plates were washed to retrieve adults for bleach/NaOH synchronization. Eggs were incubated 24 h at 23°C in M9 worm buffer with shaking at 300 rpm, and L1 larvae were transferred to NGM + OP50 plates at 23°C to produce sterile adults. Young adult worms were washed from agar plates and incubated 24 h in liquid S medium with heat-killed OP50 as a food source and 100 μg/ml kanamycin to remove any adhered or internalized OP50, producing microbe-free 2-day adults for colonization.

Bacteria were grown as described for *in vitro* competition experiments, resuspended to uniform densities (∼10^9^ cells/ml) in liquid S medium, and mixed to obtain feeder cultures containing 90% TEM-20 and 10% TEM-52. Synchronized adult worms were colonized by feeding for 36 h in 1-ml bacterial cultures in 24-well plates, which were covered with Breathe-Easy transparent membranes (Diversified Biotech) to allow gas exchange and loosely covered with foil to protect cultures from light. After colonization, worms were washed to remove external bacteria, then transferred to fresh 24-well plates in 1-ml liquid cultures of S medium containing heat-killed OP50 as a food source and different concentrations of cefotaxime (0–0.8 μg/ml) for competition. After 20-h incubation with cefotaxime, worms were washed and mechanically disrupted by grinding in 25 μl M9 worm buffer + 0.1% Triton X-100 using a Kimble Kontes motorized pestle. The resulting bacterial suspension was diluted in M9 worm buffer and plated on LB agar. Colony-forming units per worm were determined for each bacterial strain by counting YFP and CFP colonies after 48 h.

### Evolution

For evolution experiments, we started with TEM-19, TEM-20, and the A42G mutant of TEM-17. All strains were exposed to four antibiotic concentrations, and for each antibiotic condition, six independent populations were evolved. Every day, we diluted 1:225 the evolving cultures to new media with fresh antibiotic. After 13 days, scMICs of all cultures were measured and the β-lactamase genes were sequenced.

## Supporting Information

**Supplementary Information**

**Review Process File**
