# Supplementary material for: Isolated cell behavior drives the evolution of antibiotic resistance
Source: Mol Syst Biol. 2015 Jul 30;11(7):822. doi: 10.15252/msb.20145888 (PMC4547850; doi:10.15252/msb.20145888)
Supplement: Supplementary file 1 [file msb0011-0822-sd1.pdf]

Supplemental Information for *Isolated cell behavior drives the evolution of antibiotic resistance*

Tatiana Artemova, Ylaine Gerardin, Carmel Dudley, Nicole M Vega, Jeff Gore

Contents

1 Model **1**
1.1 Model description . . . 1
1.2 Solutions in various limits . . . 3
1.2.1 Death and growth time . . . 3
1.2.2 $a_{out}(t = 0) \ll K_M$ . . . 4
1.2.3 $a_{out}(t = 0) \gg K_M$ . . . 4
1.3 Generality of scMIC: growth rate as a function of internal antibiotic concentration . 5
1.4 Small cell densities . . . 5
1.5 Parameter values . . . 6

2 The model with the enzyme degradation **6**

3 Summary of all strains used in the study **7**

4 Sequencing summary **7**

5 Supplementary figures **9**

1 Model

1.1 Model description

To model the growth of a single strain, we used the following system of equations.

$$\begin{cases} \frac{\mathrm{d}a_{out}(t)}{\mathrm{d}t} = -Cn(t)(a_{out}(t) - a_{in}(t)) - y(t)\frac{V_{max}a_{out}(t)}{a_{out}(t)+K_M} \\ \frac{\mathrm{d}n(t)}{\mathrm{d}t} = \begin{cases} \gamma_g n(t)(1 - \frac{n(t)}{n_{max}}), & a_{in} < scMIC_{Dh5\alpha} \\ \gamma_d n(t), & a_{in} \geq scMIC_{Dh5\alpha} \end{cases}, \\ \frac{\mathrm{d}y(t)}{\mathrm{d}t} = \begin{cases} 0, & a_{in} < scMIC_{Dh5\alpha} \\ -\frac{dn(t)}{dt}, & a_{in} \geq scMIC_{Dh5\alpha} \end{cases}, \\ C(a_{out}(t) - a_{in}(t)) = \frac{V_{max}a_{in}(t)}{a_{in}(t)+K_M} \end{cases}, \tag{1}$$

Where
$a_{out}(t)$ – antibiotic concentration in the well (outside the cell);
$a_{in}(t)$ – antibiotic concentration inside the cell;
$n(t)$–cell density (OD);
$y(t)$ – cell density of dead (lysed) cells (OD);
C-diffusion parameter;
V_{max}, K_M-Michaelis-Menten parameters for beta-lactamase;
γ_g-growth rate;
γ_d-death rate;
$scMIC_{Dh5\alpha}$-scMIC of Dh5a;
n_{max}-saturation OD.

The first equation describes two mechanisms by which the antibiotic concentration $a_{out}(t)$ in the well decreases: antibiotic can diffuse inside a cell or can be degraded by the beta-lactamase in the media released by the dead lysed cells. The second equation describes the dynamics of cell density $n(t)$ as a function of the antibiotic concentration inside the cell (in the periplasmic space): the cells grow logistically when the concentration in the periplasm is lower some value and die and lyse exponentially when the antibiotic concentration exceeds this value. The third equation describes the time evolution of the density of the dead and lysed cells $y(t)$: whenever antibiotic concentration exceeds the scMIC of DH5a, any change in a cell density $n(t)$ is due to the cell death, therefore the density of lysed cells $y(t)$ increases by the same amount as by which the cell density $n(t)$ decreases; there is no change in the density of lysed cells $y(t)$ when periplasmic concentration of antibiotic $a_{in}(t)$ is low enough for cells to divide. The last equation describes the balance between the influx of antibiotic from the environment to periplasm of the cell and enzymatic inactivation of antibiotic in the periplasm [6].

The OD units for cell density correspond to OD600 - absorbance or optical density at 600 nm wave length light of 1 cm-wide sample of the cell culture. The OD of 1 corresponds to $4 \cdot 10^8$ CFU per ml.

For competition experiments, we use the following system of equations:

$$\begin{cases} \frac{\mathrm{d}a_{out}(t)}{\mathrm{d}t} = -Cn^{(1)}(t)(a_{out}(t) - a_{in}^{(1)}(t)) - y^{(1)}(t)\frac{V_{max}^{(1)}a_{out}(t)}{a_{out}(t)+K_M^{(1)}} - Cn^{(2)}(t)(a_{out}(t) - a_{in}^{(2)}(t)) - y^{(2)}(t)\frac{V_{max}^{(2)}a_{out}(t)}{a_{out}(t)+K_M^{(2)}} \\ \frac{\mathrm{d}n^{(1)}(t)}{\mathrm{d}t} = \begin{cases} \gamma_g n^{(1)}(t)(1-\frac{n^{(1)}(t)+n^{(2)}(t)}{n_{max}}), & a_{in}^{(1)} < scMIC_{Dh5\alpha} \\ -\gamma_d n^{(1)}(t), & a_{in}^{(1)} \geq scMIC_{Dh5\alpha} \end{cases}, \\ \frac{\mathrm{d}n^{(2)}(t)}{\mathrm{d}t} = \begin{cases} \gamma_g n^{(2)}(t)(1-\frac{n^{(1)}(t)+n^{(2)}(t)}{n_{max}}), & a_{in}^{(2)} < scMIC_{Dh5\alpha} \\ -\gamma_d n^{(2)}(t), & a_{in}^{(2)} \geq scMIC_{Dh5\alpha} \end{cases}, \\ \frac{\mathrm{d}y^{(1)}(t)}{\mathrm{d}t} = \begin{cases} 0, & a_{in}^{(1)} < scMIC_{Dh5\alpha} \\ -\frac{dn^{(1)}(t)}{dt}, & a_{in}^{(1)} \geq scMIC_{Dh5\alpha} \end{cases}, \\ \frac{\mathrm{d}y^{(2)}(t)}{\mathrm{d}t} = \begin{cases} 0, & a_{in}^{(2)} < scMIC_{Dh5\alpha} \\ -\frac{dn^{(2)}(t)}{dt}, & a_{in}^{(2)} \geq scMIC_{Dh5\alpha} \end{cases}, \\ C(a_{out}(t) - a_{in}^{(1)}(t)) = \frac{V_{max}^{(1)}a_{in}^{(1)}(t)}{a_{in}^{(1)}(t)+K_M^{(1)}} \\ C(a_{out}(t) - a_{in}^{(2)}(t)) = \frac{V_{max}^{(2)}a_{in}^{(2)}(t)}{a_{in}^{(2)}(t)+K_M^{(2)}} \end{cases} \tag{2}$$

The system of equations 2 is essentially 1 for two different cell types with densities $n^{(1)}(t)$ and $n^{(2)}(t)$, periplasmic antibiotic concentrations $a_{in}^{(1)}(t)$ and $a_{in}^{(2)}(t)$, densities of lysed cells $y^{(1)}(t)$ and $y^{(2)}(t)$. These two cell types share external environment which can be seen in the system of equations in two ways: $a_{out}(t)$ is the same for both cell types and logistic growth part ensures that the carrying capacity of nutrients is shared by two types evenly ($\gamma_g n^{(1)}(t)(1-\frac{n^{(1)}(t)+n^{(2)}(t)}{n_{max}})$ and $\gamma_g n^{(2)}(t)(1-\frac{n^{(1)}(t)+n^{(2)}(t)}{n_{max}})$ terms).

1.2 Solutions in various limits

In order to get some intuition about model prediction for the inoculum effect curve of a single strain, we will consider the limits of small and large initial antibiotic concentration relative to the K_M of the enzyme. In section 1.2.1, we will derive the expression for the duration of the death phase of the bacterial growth for which $a_{out}(t=0) = MIC$. In sections 1.2.2 and 1.2.3, we will derive the expression for the inoculum effect curve in the limits of low and high initial antibiotic concentrations $a_{out}(t=0)$ respectively.

1.2.1 Death and growth time

In our model, the cells either grow exponentially or die exponentially. Assuming that initial cell density is n_0 and final cell density at $t_{20} = 20\,hours$ is n_f (which is fixed in the MIC experiment), we can write the following system of linear equations on the time intervals when the culture dies t_{death} and the time interval when the culture grows t_{growth}:

$$\begin{cases} t_{death} + t_{growth} = t_{20} \\ \gamma t_{growth} - \gamma_d t_{death} = ln(n_f/n_0) \end{cases}, \tag{3}$$

with the solution for t_{death}

$$t_{death} = (\gamma t_{20} - ln(\frac{n_f}{n_0}))/(\gamma_d + \gamma). \tag{4}$$

1.2.2 $a_{out}(t=0) \ll K_M$

Then $a_{out} \ll K_M$ and$a_{in} \ll K_M$ for all t, and

$$a_{out} = a_{in}(\frac{V_{max}}{CK_M} + 1). \tag{5}$$

Next, while $a_{out} > scMIC$ (the same as $a_{in} > scMIC_{Dh5\alpha}$),

$$n(t) = n_0 \exp(-\gamma_d t), \tag{6}$$

$$y(t) = n_0(1 - \exp(-\gamma_d t)). \tag{7}$$

Therefore,

$$\frac{da_{out}}{dt} = -n_0(1 - \exp(-\gamma_d t))\frac{V_{max}}{K_M}a_{out} - n_0 \exp(-\gamma_d t)(\frac{\frac{V_{max}}{K_M}}{1 + \frac{V_{max}}{CK_M}})a_{out} \tag{8}$$

with the solution

$$a_{out} = a_0 \exp(-\frac{n_0 V_{max}(CK_M t + \frac{\exp(-\gamma_d t)V_{max}}{\gamma_d} + V_{max}t)}{K_M(CK_M + V_{max})}). \tag{9}$$

Plugging in $a_0 = MIC$, $a(t = t_{death}) = scMIC$,

$$ln(\frac{MIC}{scMIC}) = \frac{n_0 V_{max}((CK_M + V_{max})(\gamma t_{20} - ln(\frac{n_f}{n_0}))/(\gamma_d + \gamma) + \frac{\exp(-\gamma_d(\gamma t_{20} - ln(\frac{n_f}{n_0}))/(\gamma_d+\gamma))V_{max}}{\gamma_d})}{K_M(CK_M + V_{max})}. \tag{10}$$

For the parameter values that we have, we can ignore the exponent at the right-hand side and logarithmic dependence on n_0:

$$MIC \propto scMIC \exp(\frac{n_0 V_{max}}{K_M}\frac{\gamma t_{20}}{\gamma_d + \gamma}). \tag{11}$$

Thus, at low initial antibiotic concentrations ($MIC \ll K_M$), MIC increases exponentially with the increase of n_0.

1.2.3 $a_{out}(t=0) \gg K_M$

Typical $scMIC \leq K_M$, so the initial antibiotic concentration is much higher than $scMIC$ and the cells initially die. In order for initial antibiotic concentration to be an MIC, the death phase should be significantly long (otherwise, the regrowth will happen faster than in 20 hours). This fact together with an observation that $a_{in} \leq a_{out}$ allows us to disregard the antibiotic hydrolysis inside the cell and only consider hydrolysis outside:

$$\frac{da_{out}}{dt} = -n_0(1 - \exp(-\gamma_d t))V_{max} \tag{12}$$

note that we assume $a_{out} \gg K_M$ for all t, which is not the case when antibiotic is almost completely hydrolyzed.

$$a_{out} = a_0 - n_0 V_{max}(t + \frac{\exp(-\gamma_d t)}{\gamma_d}) \tag{13}$$

Whenever a_{out} becomes comparable to K_M, a_{out} starts to be broken exponentially in time. However, in the limit of high enough a_{out}, this time of exponential hydrolysis will be much smaller than the time of linear hydrolysis 13.

Plugging in 13 $a_0 = MIC$, $a(t = t_{death}) = scMIC$,

$$scMIC = MIC - n_0 V_{max}(\gamma t_{20} - ln(\frac{n_f}{n_0}))/(\gamma_d + \gamma). \tag{14}$$

Ignoring the logarithmic term,

$$MIC \propto scMIC + n_0 V_{max} \frac{\gamma t_{20}}{\gamma_d + \gamma}. \tag{15}$$

Thus, at high initial antibiotic concentrations ($MIC \gg K_M$), MIC increases linearly with the initial cell density n_0.

1.3 Generality of scMIC: growth rate as a function of internal antibiotic concentration

In the derivations above, we assume that the growth rate is a step function of antibiotic concentration: $\gamma(a_{out}) = \gamma_g$ for $a_{out} < scMIC$ and $\gamma(a_{out}) = -\gamma_d$ for $a_{out} > scMIC$. However, $\gamma(a_{out})$ can be any weakly decreasing function. The concept of $scMIC$ is general and useful for various functions $\gamma(a_{out})$. The following statements hold as long as resistance mechanism is cooperative:

a. The general scaling of MIC^* as a function of initial cell density are independent of the exact functional form of $\gamma(a_{out})$:

   1. MIC^* scales exponentially with the initial cell density when smaller than K_M;
   2. MIC^* scales linearly with the initial cell density when larger than K_M.

b. $scMIC$ is well-defined because the inoculum effect curve asymptotically approaches a limit at small initial cell densities.

1.4 Small cell densities

The term "small (initial) cell densities" that we are using corresponds to the dilute conditions, when the cooperative part of the resistance is very weak. From equation11, the dilution condition is as follows:

$$\frac{n_0 V_{max}}{K_M} \frac{\gamma t_{20}}{\gamma_d + \gamma} \ll 1 \tag{16}$$

which leads to

$$n_0 \ll \frac{K_M}{V_{max} t_{20}} \tag{17}$$

which under conservative assumptions (see 1.5) of $K_M = 10 \mu g/ml$, $V_{max} = 10^4 \mu g/ml$ per hour per OD results in the following condition:

$$n_0 \ll 2 \cdot 10^4 cells/ml \tag{18}$$

1.5 Parameter values

Strain independent:

Parameter	Value	Justification
C	23.4 per hour per OD	Inoculum curve fit
γ	1.4 per hour	Experimentally measured
γ_d	2 per hour	Experimentally measured
$MKC_{Dh5\alpha}$	0.03 $\mu g/ml$	Experimentally measured
n_{max}	3 OD	Experimentally measured

Supplementary Table S1: Parameter values for fits in the main text.

Strain dependent:

Parameter	Value	Justification
TEM-20 V_{max}	8400 $\mu g/ml$ per hour per OD	Inoculum curve fit
TEM-52 V_{max}	78000 $\mu g/ml$ per hour per OD	Inoculum curve fit
TEM-20 K_M	17.28 $\mu g/ml$	Inoculum curve fit
TEM-52 K_M	16.56 $\mu g/ml$	Inoculum curve fit

Supplementary Table S2: Parameter values for fits in the main text.

While C may seem to be another parameter to the inoculum curves fit, it has a constraint that it should be the same for several inoculum effect curves. Thus, every inoculum effect curve except for one has two free parameters in their model fits.

2 The model with the enzyme degradation

While the simple model presented in 1.1 explains qualitatively the behavior of the system at low antibiotic concentrations, it fails to explain some properties of the system at high antibiotic concentrations. There are two major discrepancies:

a. Inoculum effect curve, high cell densities and high MIC^*s. The data points are not only always lower than the model prediction, but also suggest different scaling of MIC^* as a function of initial cell density than the model.

b. Competition data, high antibiotic concentrations. The data suggests that there is a second peak of selection for the more resistant strain at high antibiotic concentration, while the simple model suggests that above the $scMIC$ of the more resistance strain, selection level relaxes to some level with no dips or peaks.

The discrepancies above happen in different experiments under similar conditions - at high initial antibiotic concentrations. This is why it might be the case that they happen for the same reason. We have considered several ways in which our model can be modified, out of which introducing beta-lactamase degradation turned out to be the most promising one.

The enzyme degradation may happen on its own and because of the reversible substrate-induced inactivation[1]. In the model below, we make two assumptions:

a. Different enzymes have different degradation rate in the absence of antibiotic.

b. The degradation rate of an enzyme is a linear function of antibiotic concentration - the higher antibiotic concentration, the higher the degradation rate.

Generally, as long as inhibition changes V_{max} as a function of antibiotic concentration, the scaling of the inoculum effect curve at high antibiotic concentrations should become sublinear. That means that if the inhibition is accounted for it takes longer to inactivate the antibiotic to the level of $scMIC$ than without inhibition and the effect of inhibition is larger at high antibiotic concentrations, which makes the selection increase the second time at high antibiotic concentrations.

Given two observations above, we constructed a model, which incorporates the degradation rate of beta-lactamase, linearly proportional to the cefotaxime concentration.

$$\begin{cases} \frac{\mathrm{d}a_{out}(t)}{\mathrm{d}t} = -Cn(t)(a_{out}(t) - a_{in}(t)) - y(t)\frac{V_{max}a_{out}(t)}{a_{out}(t)+K_M} \\ \frac{\mathrm{d}n(t)}{\mathrm{d}t} = \begin{cases} \gamma_g n(t)(1 - \frac{n(t)}{n_{max}}), & a_{in} < scMIC_{Dh5\alpha} \\ \gamma_d n(t), & a_{in} \geq scMIC_{Dh5\alpha} \end{cases}, \\ \frac{\mathrm{d}y(t)}{\mathrm{d}t} = \begin{cases} -\alpha y(t)a_{out}(t), & a_{in} < scMIC_{Dh5\alpha} \\ -\frac{dn(t)}{dt} - \alpha y(t)a_{out}(t), & a_{in} \geq scMIC_{Dh5\alpha} \end{cases}, \\ C(a_{out}(t) - a_{in}(t)) = \frac{V_{max}a_{in}(t)}{a_{in}(t)+K_M} \end{cases},$$

where α the enzyme degradation rate per unit of antibiotic concentration. Figure S7 shows the fits of the inoculum effect curves and the model prediction for competition experiments for $\alpha_{TEM-20} = 0.003\,(hour\,\frac{\mu g}{mL})^{-1}$ and $\alpha_{TEM-52} = 0.001\,(hour\,\frac{\mu g}{mL})^{-1}$. The other parameters stay the same as in the main text fits.

3 Summary of all strains used in the study

All the strains used in the study are identical except for the beta-lactamase gene and absent/present fluorescent protein producing plasmids. These plasmids may or may not be present in the strains depending on whether the experiment requires fluorescent labeling. The table below summarizes various strain types based on their beta-lactamase gene and relate the version of the gene to TEM-1.

Name(s) used in text	Mutations from TEM-1	scMIC in cefotaxime
reference strain, TEM-20	M182T,G238S	0.65
mutant strain, TEM-52	E104K,M182T,G238S	8
A42G mutant of TEM-20	A42G,M182T,G238S	1.78
TEM-15	E104K,G238S	1.59
TEM-19	G238S	0.22
A42G mutant of TEM-17	A42G,E104K	0.11

Supplementary Table S3: scMIC's of the strains mentioned.

4 Sequencing summary

Below is the summary of the mutations observed in the end of the evolution experiment (Fig 1c and S4).

Starting strain	Evolving antibiotic concentration, $\mu g/mL$	Final scMIC, $\mu g/mL$	Mutation observed	Comments
TEM-19	0.06	0.25	Promoter AACCCTGAT->AAACCTGAT, L12F	Cannot find any information
TEM-19	0.25	0.5	observed synonymous mutation at position 20 GCC->GCT	
TEM-19	0.5	1	A184V	A known mutation which is present in TEM-116, TEM-157, TEM-162, TEM-181, TEM-187 and TEM-119 [2].
TEM-20	0.7	0.7	Promoter AACCCTGAT->AAACCTGAT	
TEM-20	0.17	0.7	AACCCTGAT->AAACCTGAT 50-50	
A42G mutant of TEM-17	0.25	5.7	G238A; L12F	G238A has been constructed before, demonstrated increase in k_{cat} and decrease in K_M[3]. Some mutations at 12th position have been observed, but not to F [4].
A42G mutant of TEM-17	0.25	0.5	G238S	This is a well-established mutation known to increase resistance to cefotaxime. [5]
A42G mutant of TEM-17	0.45	4	G238S	This is a well-established mutation known to increase resistance to cefotaxime.[5]

Supplementary Table S4: Evolved strains beta-lactamase sequencing summary

5 Supplementary figures

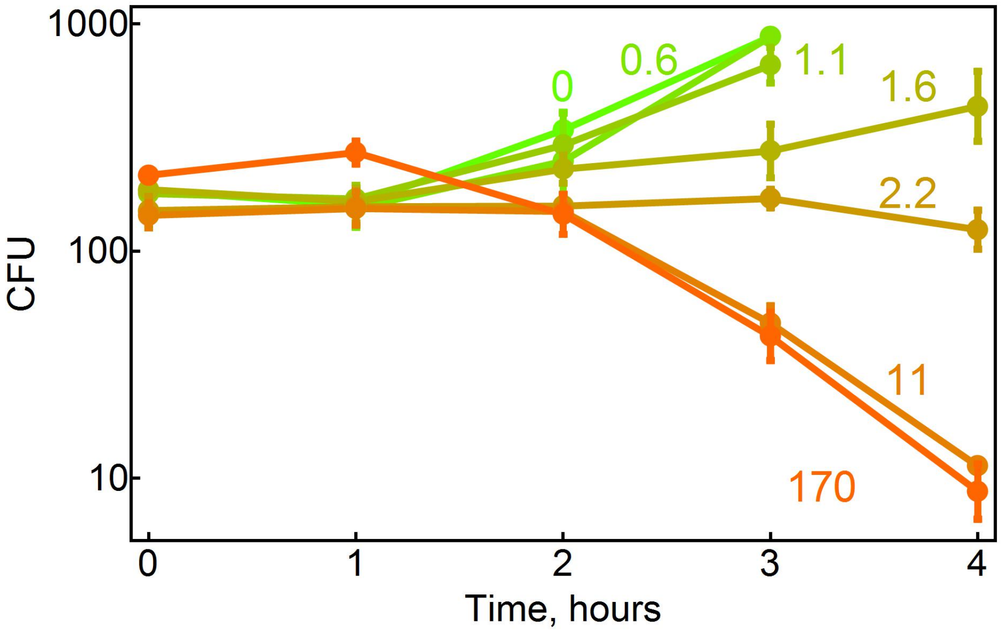

Supplementary Figure S1: Growth rate can be modeled as a step function of external antibiotic concentration. The transition between exponential growth with the highest growth rate and exponential death with the highest death rate is relatively sharp. Different colors correspond to different antibiotic concentration. A42G mutant of TEM-20 is used, the measured scMIC of this strain is 1.6 $\mu g/mL$, the measured MIC is 64 $\mu g/mL$. For this figure, the bacterial were cultured in 50 mL flasks and at the measurement time point some dilution of bacteria were plated. The error bars correspond to the square root of the CFU.

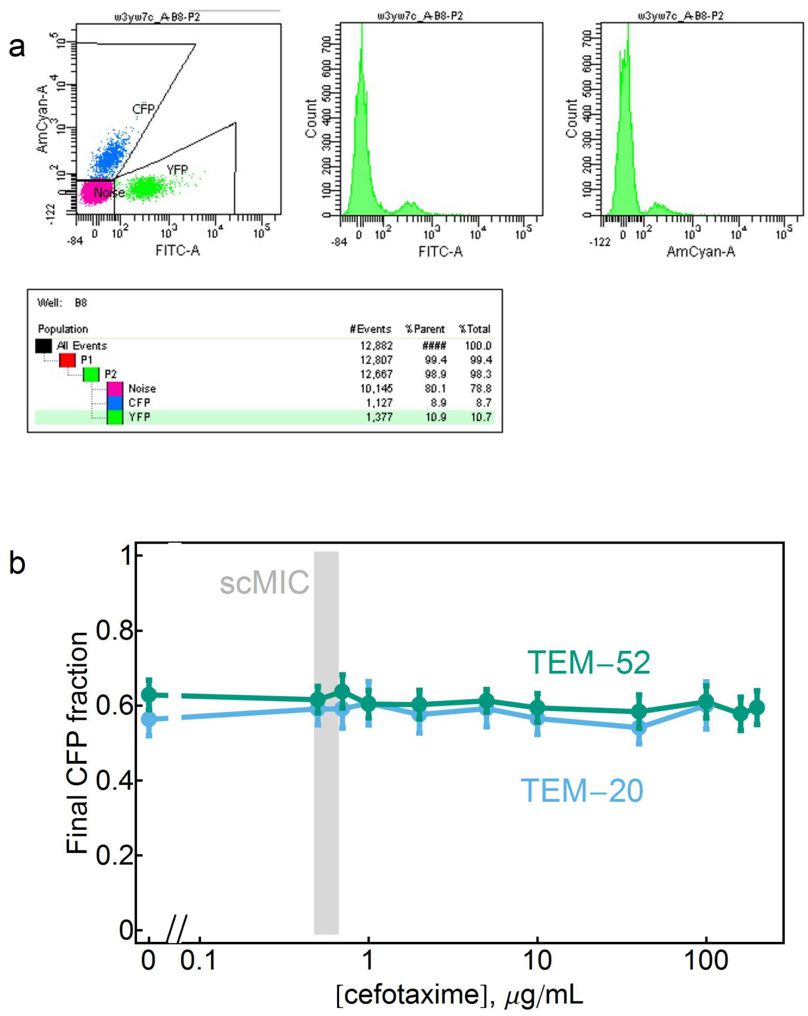

Supplementary Figure S2: Flow cytometry fraction measurements (a) YFP (x-axis) and CFP (y-axis) signals on one plot and histograms of the YFP and CFP counts. (b) TEM-20 and TEM-52 strains of different colors competing. The fraction of CFP variant of the strain stays flat as a function of antibiotic concentration. This figure proves that selection patterns observed in the main text are not due to the influences of cefotaxime presence on kanamycin plasmids.

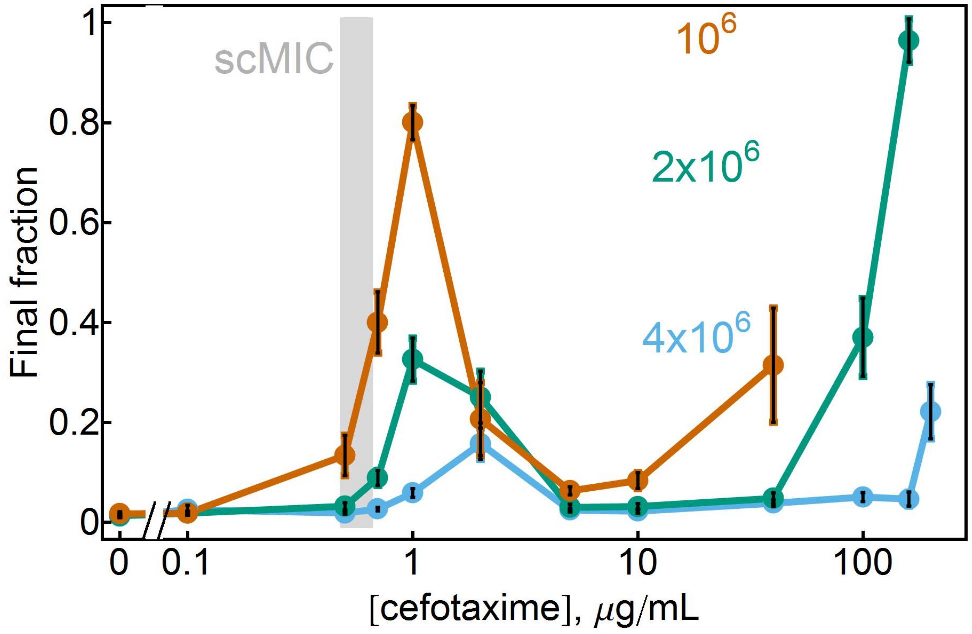

Supplementary Figure S3: In competition of TEM-20 and A42G mutant of TEM-20, selection for the mutant begins at the scMIC, not MIC*, of TEM-20. For most of the data points, the mean values for 8 different cultures with different coloring of the strains are presented. The error bars are the standard error of the mean. The gray bars correspond to the scMIC value of TEM-20. The scMIC and MIC* of TEM-20 are 0.8 $\mu g/mL$ and 14.3 $\mu g/mL$ correspondingly, the MKC and MIC* of A42G mutant of TEM-20 are 1.59 $\mu g/mL$ and 64 $\mu g/mL$ correspondingly.

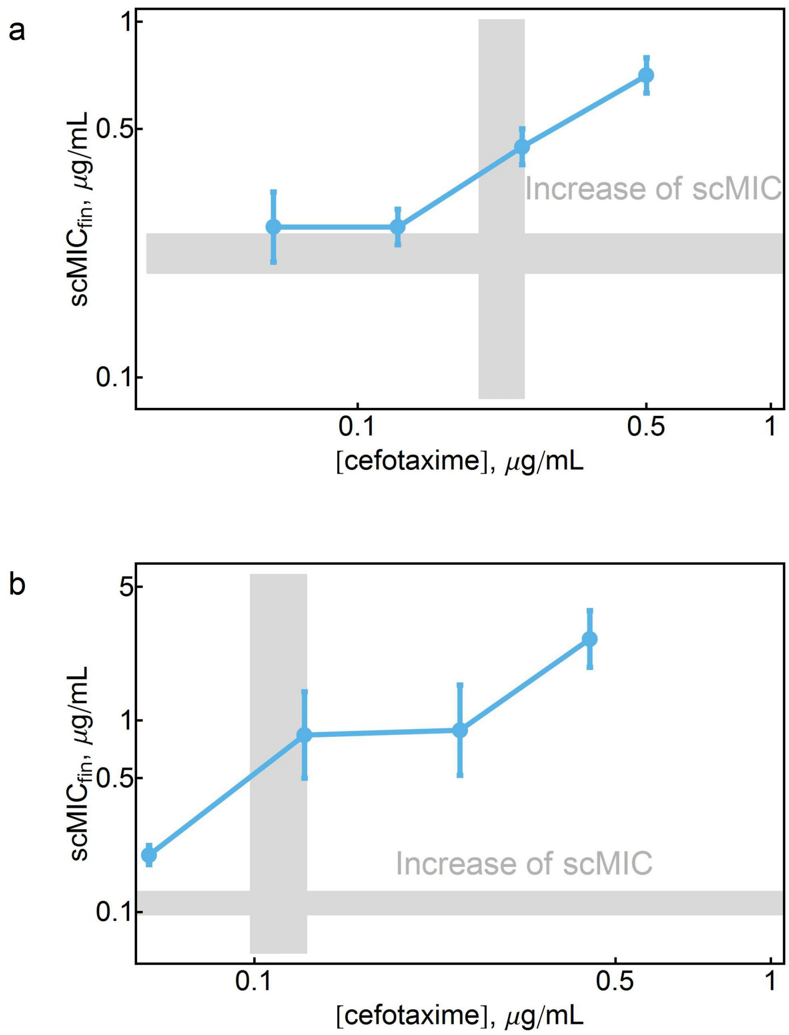

Supplementary Figure S4: Laboratory evolution experiments of (a) TEM-19 and (b) A42G mutant of TEM-17 confirm that increase of resistance evolves in antibiotic concentrations equal to and larger than the scMIC. Plotted is the scMIC measured after 13 days (~100 generations) versus the concentration of cefotaxime the strains were evolved at. The error bars are the standard errors of the mean of six independent evolved populations. The gray bars correspond to the initial scMIC values.

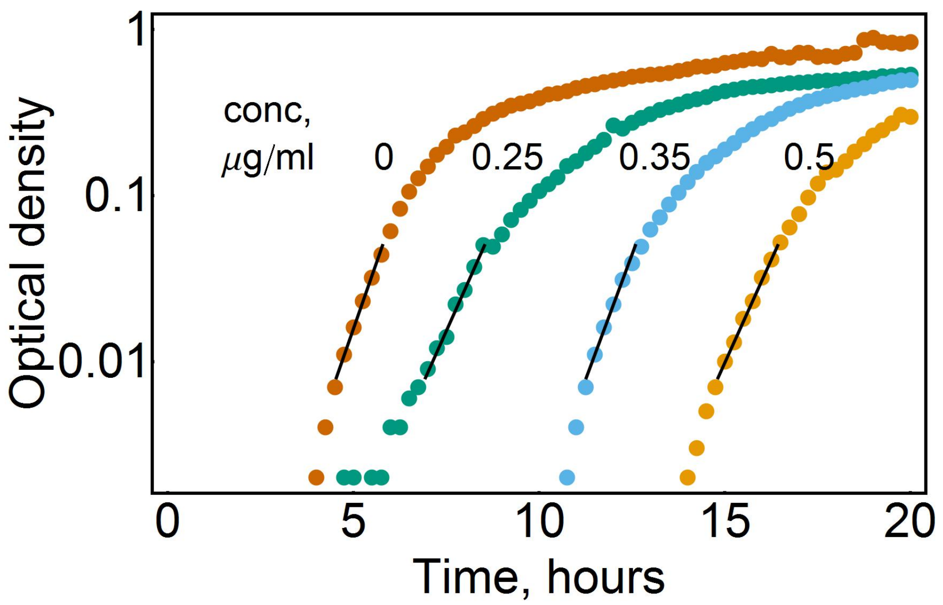

Supplementary Figure S5: Growth curves of TEM-20 in different antibiotic concentrations. With the increase of antibiotic concentration, the slope of the growth curves does not change, while the time to reach some optical density increases. This is a result of the cooperative growth: the cells first cooperatively hydrolyze cefotaxime and then grow with maximal division rate.

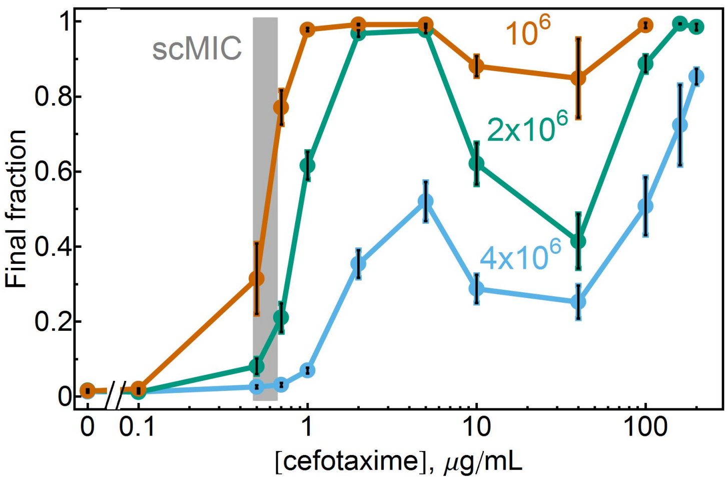

Supplementary Figure S6: The experimental data for competition experiments, with a 1% initial fraction of the mutant strain. At the scMIC of the reference strain, the final fraction of the mutant starts to increase, indicating that selection for the more resistant mutant starts near the scMIC. Different colors correspond to different initial cell densities (labeled in CFU/ml). The error bars are the standard error of the mean (n = 9 – 10 for most data points). The gray bar corresponds to the scMIC of the reference strain. For the model, parameter values are provided in section 1.5.

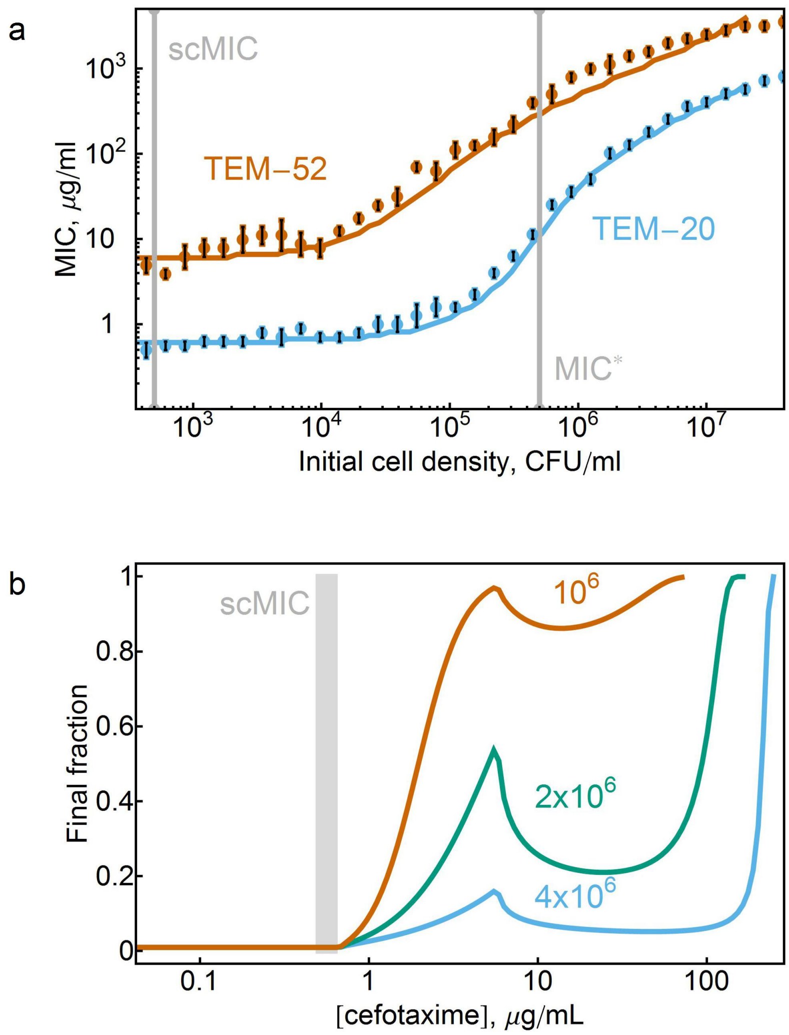

Supplementary Figure S7: The model with the enzyme-cefotaxime irreversible binding explains the inoculum effect and also the selection patterns at high antibiotic concentrations. (a) The fits of the inoculum effect curves of TEM-20 and TEM-52. The error bars are the maximum of a discretization error and the standard error of the mean of three measurements. (b) The model prediction for competition experiments, with a 1% initial fraction of TEM-52. At high antibiotic concentrations, the second selection peak appears. The parameter values used can be found in sections 1.5 and 2. Different colors correspond to different initial cell densities (labeled in CFU/ml). The gray bar corresponds to the scMIC of TEM-20.

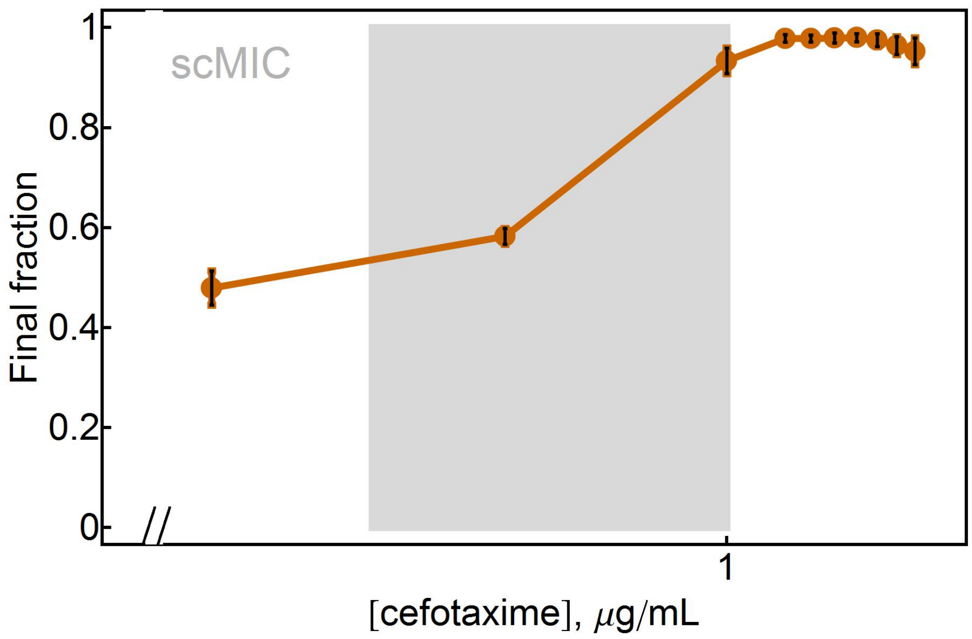

Supplementary Figure S8: Selection favors an increase of scMIC not MIC*. The competition experiment of TEM-15 and the A42G mutant of TEM-19 (initial fraction plotted as horizontal line, initial cell density 5x10^5 cells/ml). TEM-15 has a higher scMIC (1.78 $\mu g/mL$ vs 1.26 $\mu g/mL$), while both strains have similar MIC* values (18 $\mu g/mL$ vs 20 $\mu g/mL$, which are statistically indistinguishable, because the antibiotic dilution factor is $\sqrt{2}$). For cefotaxime concentrations above the scMIC of the A42G mutant of TEM-19, the TEM-15 strain is selected for, indicating that selection maximizes the scMIC rather than the MIC*. Error bars are the standard errors of the mean of 4 independent populations.

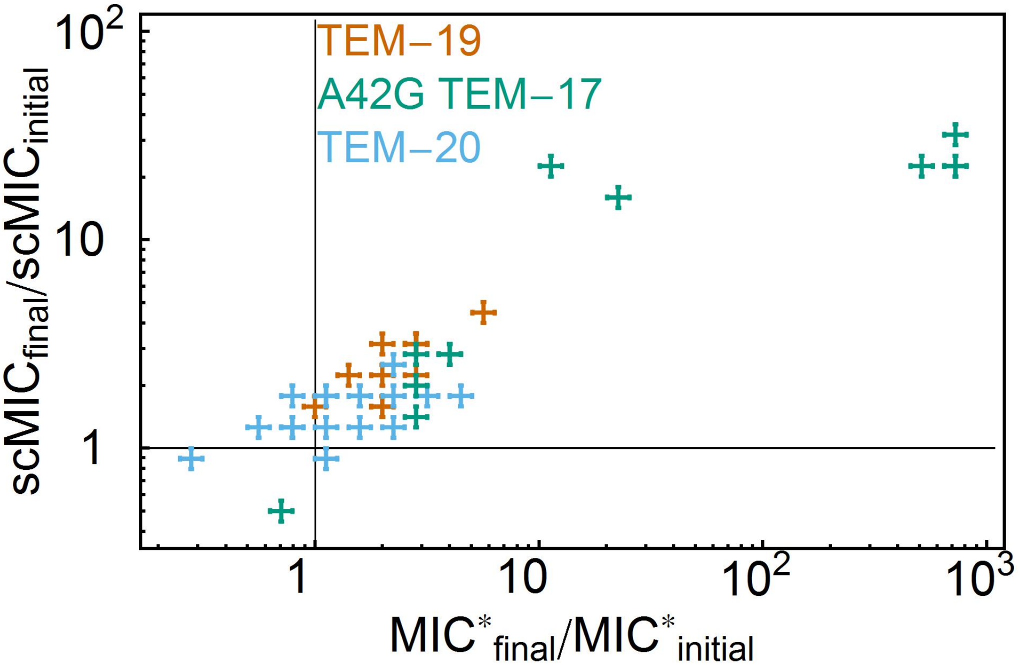

Supplementary Figure S9: Sometimes an increase in scMIC is accompanied by the decrease in MIC*. The scMIC vs MIC* values of the evolved cultures as a fraction of the initial scMIC or MIC* values are presented. Only data for the cultures evolved at concentrations equal to or greater than the scMIC of the initial strain is shown. Different colors correspond to different strains evolved. The data presented is the same as in Fig. 1c and S4.

References

[1] R.A. Bonomo. *Enzyme-mediated resistance to antibiotics: mechanisms, dissemination, and prospects for inhibition.* Amer Society for Microbiology, 2007.

[2] K. Bush and G. Jacoby. Nomenclature of tem beta-lactamases. *Journal of Antimicrobial Chemotherapy*, 39(1):1–3, 1997.

[3] C. Cantu III and T. Palzkill. The role of residue 238 of tem-1 β-lactamase in the hydrolysis of extended-spectrum antibiotics. *Journal of Biological Chemistry*, 273(41):26603–26609, 1998.

[4] S. Demanèche, H. Sanguin, J. Poté, E. Navarro, D. Bernillon, P. Mavingui, W. Wildi, T.M. Vogel, and P. Simonet. Antibiotic-resistant soil bacteria in transgenic plant fields. *Proceedings of the National Academy of Sciences*, 105(10):3957–3962, 2008.

[5] D.M. Weinreich, N.F. Delaney, M.A. DePristo, and D.L. Hartl. Darwinian evolution can follow only very few mutational paths to fitter proteins. *science*, 312(5770):111–114, 2006.

[6] Willy Zimmermann and Armel Rosselet. Function of the outer membrane of escherichia coli as a permeability barrier to beta-lactam antibiotics. *Antimicrobial agents and chemotherapy*, 12(3):368–372, 1977.
