# Supplementary material for: Isolated cell behavior drives the evolution of antibiotic resistance
Source: Mol Syst Biol. 2015 Jul 30;11(7):822. doi: 10.15252/msb.20145888 (PMC4547850; doi:10.15252/msb.20145888)
Supplement: Supplementary file 2 [file msb0011-0822-sd2.pdf]

Isolated cell behavior drives the evolution of antibiotic resistance

Tatiana Artemova, Ylaine Gerardin, Carmel Dudley, Nicole M. Vega, Jeff Gore

Corresponding author: Jeff Gore, MIT

Review timeline:

Submission date:	03 November 2014
Editorial Decision:	12 December 2014
Revision received:	15 April 2015
Editorial Decision:	11 May 2015
Revision received:	16 June 2015
Accepted:	2 July 2015

Editor: Maria Polychronidou

Transaction Report:

(Note: With the exception of the correction of typographical or spelling errors that could be a source of ambiguity, letters and reports are not edited. The original formatting of letters and referee reports may not be reflected in this compilation.)

1st Editorial Decision 12 December 2014

Thank you again for submitting your work to Molecular Systems Biology. We have now heard back from the three referees who agreed to evaluate your manuscript. As you will see from the reports below, the referees acknowledge that you address a potentially interesting topic. However, they raise a series of concerns, which should be carefully addressed in a revision of the manuscript.

Without repeating all the points listed below, some of the more fundamental issues are the following:
- Additional modeling and experimental analyses are required in order to better support the conclusion that the scMIC is a good metric for predicting selection and to demonstrate its more general relevance, beyond the context analyzed here.
- Experimental analysis of the mechanisms related to increased scMIC would enhance the completeness of the work.
- The clinical relevance and potential applications of the scMIC need to be addressed.
- Moreover, several of the comments of the reviewers refer to technical concerns and to the need to clarify several points throughout the manuscript.
- Finally the reviewers suggest replacing scMIC with a more accurate term, since the scMIC is not measured at the single cell level.

Reviewer #1:

This is an interesting but not very impactful study of the relationship of clinical MIC protocols to a more accurate "single cell MIC" (scMIC) to fitness. The authors show that the population MIC is a

© European Molecular Biology Organization

poor proxy to fitness since it does not take into account the population density effects on beta-lactam inactivation. Based upon the scMIC they go onto to model the kinetics of the beta-lactamase on periplasmic cefotaxime concentrations to produce fits to their data. The major supposition is that the scMIC is the level at which evolution acts (lines 261-272). The paper is rather full of generalities and overstatements that are not well substantiated. For example the idea that the scMIC is the level of selection is true under certain circumstances but untrue for many others. Infections such as bacteremias, bladder infections or heart valve endocarditis do have an important population and biofilm character that cannot be ignored. Likewise in ecological contexts like soil the population matters. This same idea is stated again in lines 330-332 and is just categorically wrong.

Additionally, the authors state "Thus the MIC plays a major role in our understanding of the evolution of antibiotic resistance in bacteria." The MIC is a quick and dirty clinical metric used to determine the concentration of drug required to inhibit growth. It is defined in the CLSI and is protocol that can be readily implemented in clinical setting by clinical laboratories. As a consequence, MICs are notoriously poor proxies for fitness. Perhaps growth rates as a function of drug might be more informative but while the authors are right to question the value of MICs I think this is a bit of a strawman. To be quite frank, I don't understand why anyone would use an MIC as a proper fitness metric. This is especially egregious when you consider that beta-lactams are bacteriocidal and not bacteriostatic.

I am not sure the authors are actually measuring a "single cell" MIC when in fact they are just diluting to a very low population number. They go on to show that their measurements are in good agreement with the well known microdilution assay that many micro labs use. I also don't think it is broadly true that selection occurs at or near the MIC. In fact, selecting at the MIC for a bacteriocidal antibiotic produces no growth (that is its definition) and is not how experimental evolution is conducted. There are a growing number of papers that use sub-MIC concentrations of drug to select for adaptive mutations (Kohanski etal Mol Cell 2010 is an example but there are many).

It was not clear to me in the system of equations in the Supplement how the periplasmic concentrations of various TEM mutants was estimated. Are these mutants equally well exported to the periplasm? In competition experiments at higher densities/high drug concentrations the model has difficulty and the authors propose degradation of the enzymes in these conditions. While it might be true is there any experimental reason to think that would be linear? Wouldn't substrate binding be saturating under these conditions?

Minor points:

1. Line 51 [CITE EUGENE] in the third paragraph of the introduction. I'm guessing you missed a Shakhnovich reference.
2. Inconsistency between using "Figure" or "Fig" or "Fig.".
3. Line 130 Results section Selection, (8 g/mL vs).
4. Spell out C. elegans the first time it is used.
5. Supplemental Sequencing summary table and FigureS3, Final "MKC" should be changed to "scMIC" in the third column.
6. Supplemental Figure 8, the third sentence in the legend is a fragment.
7. Superscripts are used throughout the paper except when describing the standard cell density (5x10^5 cells/ml) for MIC* tests.
8. Figure 1c legend states that "different coloring of the strains are presented", however, I only see one color and it is not specified in the legend or figure which mutant this color represents. The full text says that this represents TEM-20 but there is inconsistency and confusion between the full text and figure legend.

Suggested alterations:
1. I strongly recommend an additional table specifying the genotypes of the different TEM strains and their MIC*. It was difficult reading the paper without that information in a specific location/ having to look for that information embedded in the text or figure legends.
2. When first introducing -lactams, it would be beneficial to clearly mention that they are bactericidal antibiotics as it allows for better understanding of later figures such as Figure S1. Otherwise, it could appear that concentration 2.2 g/mL is the lowest concentration that results in cell death because it shows a decrease in CFU.

3. I would recommend changing the colors of Figure S1 so that high drug concentrations are in red (cell death) and low drug concentrations are in green (cell growth) for a more intuitive understanding of the figure.
4. In the Sequencing summary table, it is mentioned that when evolving TEM-19 to 0.25 g/mL of drug, there is a "silent mutation at position 20 GCG -> GCT". I recommend being conservative and saying that this is an observed synonymous mutation unless it is definitely known that this mutation is also silent. Synonymous mutations can alter rate and efficiency of translation causing them to be non-silent.
5. In Figure S2, is the color of the CFP histogram intended to be blue instead of green to specify that it is CFP counts and not YFP counts?
6. In Figure S4, I highly recommend using the same scales for both the x and y-axis to provide a more honest representation of the data and allow for ease of comparison.
7. Why cite CLSI antimicrobial susceptibility testing for anaerobic bacteria when you are using E. coli, an aerobic bacterium?

Suggested Experiments: Nitrocefin Assay- -lactamases can be secreted by bacteria into the media. As a control it would be good to test the amount of TEM enzyme in your media that could also be inactivating cefotaxime and altering the steady-state levels of this drug.

Reviewer #2:

The authors sought to understand the evolution of bacterial resistance through the analysis of a novel metric, termed here scMIC. In contrast to conventional knowledge typically associating bacterial population resistance with the MIC, or the minimum inhibitory concentration, the authors argued that the single cell MIC (scMIC) would serve as a better measure of evolutionary fitness. To demonstrate this point, the authors used plasmid-encoded Bla and determined population MIC or scMIC by varying the initial cell density, where low enough cell density would remove the cooperation ability for Bla to act as a public good. The authors found that the scMIC was always at least an order of magnitude smaller than the measured MIC both in liquid culture and solid phase.

The authors then demonstrated that selection within a bacterial population begins at the antibiotic concentration corresponding to the scMIC, not the MIC, which has particular relevance for the development of resistant populations in vivo. To investigate competition of a mixed bacterial population, the authors paired beta-lactamase plasmids and introduced various antibiotic concentrations below, at or above the scMICs of each harboring strain. They investigated the idea that antibiotic between the two scMIC values of competing strains will favor the one retaining the higher resistance, but once passed both scMIC levels, neither has a fitness advantage. Experimental results support this observation. To investigate this further, the authors implemented a C. elegans model to analyze population resistance in vivo, which was consistent with in vitro experiments as well. The authors then developed a simple mathematical model to explain the observations, which accurately predicts the relationship of MIC and cell density, as well as nonlinear relationship between scMIC and antibiotic. Lastly, the authors investigate the relationship between MIC and scMIC, demonstrating that the inoculum effect plays a crucial role in the discrepancy between scMIC and MIC. The authors conclude by stating the importance of understanding the evolution of bacterial antibiotic resistance, and how this metric allows for increased understanding of selection and advantage of populations under selective antibiotic pressure.

General Comments:
Although the idea of inoculum effect is well known, this work is novel in its attempt to derive a metric that is independent of the initial cell density. The key idea underlying the scMIC is that at a sufficiently low density, the cooperative nature of antibiotic degradation is negligible. If measured at such a density, the MIC reflects what is needed to kill individual cells. As a result, this scMIC is independent of cell density. The work is interesting and overall well presented. This study is timely, given the need for improved treatment strategies, insightful, given the need to better understand how population level dynamics affect drug responses, and well thought out. It merits publication with some clarifications and revisions.

While I like the concept of scMIC, however, I have major reservations about the claim that it's the best metric to predict selection. For instance, the authors use this study to claim that scMIC is superior in order to quantify antibiotic resistance. Resistance can certainly be split in separate

categories. However, population level resistance does exist, and for that MIC may still be relevant in order to understand larger-scale phenotypes, even if resistant 'evolution' begins at the scMIC. By the nature of the definition, scMIC seems an appropriate metric when there is negligible turnover of the antibiotic. In a mixture consisting of two populations with different scMICs, the longer-term dynamics of the two populations will be highly dependent on the subsequent turnover of the antibiotic by either or both populations. In this situation, the predictive power of scMIC is likely limited. It is unclear how the authors avoided this complication in their experiments (in vitro or in vivo). It would be helpful to clarify this point by additional modeling and/or experimental analysis, or better clarify the application context of scMIC.

Other specific comments:
1. All of the beta-lactamase used were TEM plasmid-born resistance. Are they under the same promoter? Are some constitutive and others inducible by the antibiotic? This could change interpretation of the results. I assume they were all constitutive; could the authors comment on how this would change with inducible resistance? Also, what does the scMIC look like for a sensitive strain?

2. Although MIC is a potent indicator, beta-lactams actually respond more to time above MIC than concentration above MIC. Do the authors have any indication whether the time spent above the scMIC has the same effect? This is especially relevant for beta-lactams and would be interesting to see.

3. While I understand the rationale of the term single-cell MIC, it is still empirically determined with a small bacterial population (~500cells/mL). I would prefer the authors use a different term for consistency.

4. Although interesting, the density will never be so low in vivo and the MIC is still more relevant for clinical applications. Can the authors comment on how this might affect clinical interpretations of MIC? In particular, how would one go about using scMIC to guide clinical intervention? Some comments on this point would be useful.

5. An increase in scMIC vs an increase in MIC may not be a fair comparison. If one does not occur simultaneously with the other, then different mechanisms are at play and both metrics would provide valuable insight into population level resistance. As noted above, in a longer term, as the bacterial populations degrade the antibiotic (which underlies the inoculum effect), the predictive power of the scMIC for the ultimate evolution dynamics may be limited.

6. When the authors state "selection for increase scMIC", what's the mechanism for the increased scMIC? It would be useful to provide some experimental evidence (e.g. mutations underlying this increase). As noted above, selection for MIC and scMIC may occur at different levels. I presume scMIC is happening at the genetic level, meaning that population level conclusions may not be fair to draw.

Reviewer #3:

The authors of this manuscript introduce the concept of single cell MIC (scMIC), which is the MIC value measured at low (~ 500 cell/ml) cell densities. They show that in the case of the -lactam antibiotic cefotaxime, which is inoculum size dependent, the scMIC is at least one order of magnitude lower than the standard MIC measured at thousand-fold higher cell densities. They also prove that at low cell densities the MIC does not depend on the cell concentration any more, providing a more robust data. This is due to the fact that at low cell densities the cooperative behavior of the cells cannot mask the real resistance level (scMIC) of the individual cells. Therefore, the authors suggest that scMIC is a superior metric for quantifying resistance, compared to the standard MIC. They also claim that at antibiotic concentrations lower than the scMIC the applied antibiotic does not affect the fitness of the cells, and therefore selection for resistant mutants starts at concentrations close to or above the scMIC. To support this hypothesis adaptive laboratory evolution experiments were carried out in the presence of cefotaxine, as well as competition experiments with -lactam resistant E. coli strains with different resistance levels. The authors claim that based on the results from these experiments the scMIC "predicts the direction of selection and also specifies the antibiotic concentration at which selection begins to favor new mutants"

© European Molecular Biology Organization

Major concerns:
1) Previous studies (Gullberg et al., 2011, 2014; Hughes and Andersson, 2012) have shown that selection of resistant bacteria can occur at antibiotic concentrations several hundred-fold below the MIC. Specifically, Gullberg and his colleagues (Gullberg et al., 2014) have shown that antibiotic concentrations much below the MIC are sufficiently high for maintaining multidrug resistant, extended-spectrum -lactamase (ESBL) containing plasmids. They proved this for antibiotics with different mechanism of action, including those where the inoculum size dependence of the MIC is absent. In the latter case the standard MIC and the scMIC are the same.
The authors of the present manuscript themselves state that the standard MIC "may not perform well as a measure of evolutionary fitness, even where resistance is not density-dependent. For example, a recent study demonstrated that sub-MIC*levels of tetracycline, aminoglycosides, and fluoroquinolone antibiotics can select for cells carrying an antibiotic resistance plasmid". With this statement they contradict themselves because in the case of antibiotics with no inoculum size effect the scMIC and MIC do not differ from each other, which means that selection occurs below the level of scMIC.
So my major concern regards the basic prediction of the authors that at concentrations below the scMIC antibiotics do not affect the fitness of the cells, and consequently selection does not act at concentrations below the scMIC. However, the authors themselves claim in line 211 that there is a fitness effect of the antibiotic below the scMIC concentration.
2) How much the usability of the scMIC as a metric for quantifying resistance evolution can be extrapolated from cefotaxime to other antibiotics with no inoculum size effect? I think this cannot be done, because the distinction between scMIC and MIC is relevant only for resistance mechanisms governed by enzymatic inactivation of the drug. Even in the case of cefotaxime, when the resistance level provided by a -lactamase variant was low, the scMIC and MIC values were nearly the same.
In my opinion the results presented are not systematic enough, and in the above pointed aspects are contradictory as well.

1st Revision - authors' response 15 April 2015

We appreciate the careful reading that you and the referees gave to our manuscript. Attached please find a revised version which we hope addresses the questions and concerns that were raised. Below in italics are questions / comments from you and the referees, which we have responded to in plain text. To facilitate understanding of what we have changed, in many cases we provide line numbers (corresponding to the new text) of what was added to the main text. Thank you again for your consideration of our manuscript.

Thank you again for submitting your work to Molecular Systems Biology. We have now heard back from the three referees who agreed to evaluate your manuscript. As you will see from the reports below, the referees acknowledge that you address a potentially interesting topic. However, they raise a series of concerns, which should be carefully addressed in a revision of the manuscript.

Without repeating all the points listed below, some of the more fundamental issues are the following:
- Additional modeling and experimental analyses are required in order to better support the conclusion that the scMIC is a good metric for predicting selection and to demonstrate its more general relevance, beyond the context analyzed here.
- Experimental analysis of the mechanisms related to increased scMIC would enhance the completeness of the work.
- The clinical relevance and potential applications of the scMIC need to be addressed.
- Moreover, several of the comments of the reviewers refer to technical concerns and to the need to clarify several points throughout the manuscript.
- Finally the reviewers suggest replacing scMIC with a more accurate term, since the scMIC is not measured at the single cell level.
…

In response to the referees' comments we have performed new experiments, modified multiple figures for clarity, and added additional explanation to the main text. In particular, we performed

© European Molecular Biology Organization

new microscopy experiments demonstrating that the scMIC is indeed a property of single cells (whereas before we had demonstrated this by quantifying the number of single cells on agar able to grow into macroscopic colonies). We have also significantly modified the figures, as several of our central experiments / conclusions were not properly conveyed in the original version of the manuscript. To improve the clarity of presentation we have added cartoon diagrams in several figures to explain the experiment that was performed and the base outcome. These changes and responses to the referees also resulted in changes to the text, which we detail below.

As pointed out by one of the referees, in the original version of the manuscript the strain names are difficult to follow and cause unnecessary confusion. Most of the manuscript focuses on just two strains, so we decided to refer to them as the reference and mutant strain. The only exception is the last figure in which we introduce a new pair and therefore use the names of the strains. We hope that this helps avoid unnecessary confusion. In addition, in the supplementary section we include a table of strains.
We have also incorporated more experimental measurements into our estimate of the scMIC of the reference strain, leading to a slight decrease in the value from 0.8 ug/ml to 0.65 ug/ml. This new value is within our original error bar, but we believe that the new value is more accurate.
For changes regarding more specific points see the answers to the reviewers' comments below.

Reviewer #1:

This is an interesting but not very impactful study of the relationship of clinical MIC protocols to a more accurate "single cell MIC" (scMIC) to fitness. The authors show that the population MIC is a poor proxy to fitness since it does not take into account the population density effects on beta-lactam inactivation. Based upon the scMIC they go onto to model the kinetics of the beta-lactamase on periplasmic cefotaxime concentrations to produce fits to their data. The major supposition is that the scMIC is the level at which evolution acts (lines 261-272). The paper is rather full of generalities and overstatements that are not well substantiated. For example the idea that the scMIC is the level of selection is true under certain circumstances but untrue for many others. Infections such as bacteremias, bladder infections or heart valve endocarditis do have an important population and biofilm character that cannot be ignored. Likewise in ecological contexts like soil the population matters. This same idea is stated again in lines 330-332 and is just categorically wrong.

We apologize that we did not make our point about the difference between scMIC and MIC* clear. We agree that community and population level resistance is an important aspect of antibiotic resistance and we mention it in the manuscript (lines 280-282 in the original manuscript, 303-305 in the new version: 'While we have argued that the scMIC is better than the MIC* for predicting evolution, the MIC* still contains important information that will be relevant in many contexts. For instance, MIC* captures the population level resistance due to effects such as the collective inactivation of a drug.'). We are arguing that the MIC* is quantifying this community level resistance, and indeed is important clinically because it is telling us something about how high of antibiotic concentrations would be required to clear a bacterial infection at that cell density. However, we believe that even in this situation it is the scMIC that tells us the antibiotic concentration that will start selecting for mutants with increased levels of resistance (as quantified by the scMIC, because as we have shown the MIC* and the scMIC can disagree regarding which strain is more fit in the presence of the drug). We have for example demonstrated that this is true both in vitro and in a *C. elegans* model of a bacterial infection. This obviously does not prove that it will be true in all circumstances, but we believe that it is a strong demonstration of the general nature of the phenomenon. Nevertheless, we have modified the text to clarify the ways in which we believe that the scMIC and MIC* might be useful and to point out the limitations of the scMIC:
The original lines 280-283 ("While the scMIC is very important for predicting evolution, MIC* captures the resistance level of a population and can still be useful for determining proper antibiotic dosage and regimen." have been expanded to (new lines 305-312):
"While we have argued that the scMIC is better than the MIC* for predicting evolution, the MIC* still contains important information that will be relevant in many contexts. For instance, MIC* captures the population level resistance due to effects such as the collective inactivation of a drug. This population level resistance is useful for determining proper antibiotic dosing because the entire population of cells needs to be killed, and therefore the cooperative aspect of resistance cannot be ignored. It is important to stress that predicting evolution and estimating the antibiotic concentration

required to kill a population of a given size are very different questions; while the former requires understanding the costs and benefits to a single cell, the latter requires quantification of the population level resistance.”

Additionally, the authors state "Thus the MIC plays a major role in our understanding of the evolution of antibiotic resistance in bacteria." The MIC is a quick and dirty clinical metric used to determine the concentration of drug required to inhibit growth. It is defined in the CLSI and is protocol that can be readily implemented in clinical setting by clinical laboratories. As a consequence, MICs are notoriously poor proxies for fitness. Perhaps growth rates as a function of drug might be more informative but while the authors are right to question the value of MICs I think this is a bit of a strawman. To be quite frank, I don't understand why anyone would use an MIC as a proper fitness metric. This is especially egregious when you consider that beta-lactams are bacteriocidal and not bacteriostatic.
To address this concern, we softened the statements about the importance of the MIC for prediction evolution:

- Original manuscript line 41 (new line 34): ‘The MIC is sometimes’ instead of ‘The MIC is often’
- Original manuscript line 44 (new line 38): ‘Thus, the MIC plays an important role’ instead of ‘Thus, the MIC plays a major role’
- Original manuscript line 46 (new line 40): ‘However, while the MIC is sometimes’ instead of ‘However, while the MIC is often’

I am not sure the authors are actually measuring a "single cell" MIC when in fact they are just diluting to a very low population number. They go on to show that their measurements are in good agreement with the well known microdilution assay that many micro labs use.

Regarding the “single cell” question, we have performed additional experiments to confirm that the scMIC is indeed the MIC of a single cell.
Insert paragraph after original line 115 (new line 109):
While the main method that we use to quantify the scMIC does not involve conventional single cell experimental techniques, we believe that the name “single cell MIC” is accurate for the following reasons. First, experimentally we observe the disappearance of the inoculum effect as the MIC curve plateaus at low cell densities, where dilution prevents significant depletion of the total antibiotic concentration. Second, this liquid dilution method agrees with a true single cell measurement – plating at low density on agar. In this agar plating method every observed colony is a result of growth starting from a single cell, so the presence of a colony is conditioned on the survival of a single cell in a given antibiotic environment. Finally, we used microscopy to directly observe growth of single cells in a variety of antibiotic concentrations and observed qualitatively different behavior below and above the scMIC value.

I also don't think it is broadly true that selection occurs at or near the MIC. In fact, selecting at the MIC for a bacteriocidal antibiotic produces no growth (that is its definition) and is not how experimental evolution is conducted. There are a growing number of papers that use sub-MIC concentrations of drug to select for adaptive mutations (Kohanski etal Mol Cell 2010 is an example but there are many).

We agree that it is not broadly true that selection occurs at or near the MIC. Nevertheless, there are several examples in the literature where people assume that strong selection only occurs near the MIC. This assumption of selection occurring only near the MIC is expected to be true if the growth rate as a function of antibiotic concentration is a sharp Hill function (relatively common: see the answer to reviewer #3) and there is no inoculum effect (resistance mechanism does not involve degradation). In this case there would be no inoculum effect, so the scMIC and MIC* coincide and the selection starts at the MIC* (the same as scMIC).

It was not clear to me in the system of equations in the Supplement how the periplasmic concentrations of various TEM mutants was estimated. Are these mutants equally well exported to the periplasm?

The mechanistic model proposed in this paper was fitted to the inoculum effect curves of several strains. This is how we obtained the parameter values, and given the parameter values, it is possible to predict periplasmic concentration for a given outside concentration of antibiotic.

In competition experiments at higher densities/high drug concentrations the model has difficulty and the authors propose degradation of the enzymes in these conditions. While it might be true is there any experimental reason to think that would be linear? Wouldn't substrate binding be saturating under these conditions?

Our unpublished results indicate that some degradation of the enzyme occurs. The reason we only have a linear term is because we try to minimize the number of parameters used in the model. In the main text, we say "we can allow for degradation" meaning that other mechanisms are still possible. We believe it is better to avoid an extensive discussion of this issue in the main text, because this is a minor point of the paper, may be system-specific and can distract the reader from the main conclusions.

Minor points:

1. Line 51 [CITE EUGENE] in the third paragraph of the introduction. I'm guessing you missed a Shakhnovich reference.
Fixed.
2. Inconsistency between using "Figure" or "Fig" or "Fig.".
Fixed
3. Line 130 Results section Selection, (8μg/mL vs).
Fixed.
4. Spell out C. elegans the first time it is used.
Fixed.
5. Supplemental Sequencing summary table and FigureS3, Final "MKC" should be changed to "scMIC" in the third column.
Fixed.
6. Supplemental Figure 8, the third sentence in the legend is a fragment.
Fixed.
7. Superscripts are used throughout the paper except when describing the standard cell density (5x10^5 cells/ml) for MIC tests.*
Fixed.
8. Figure 1c legend states that "different coloring of the strains are presented", however, I only see one color and it is not specified in the legend or figure which mutant this color represents. The full text says that this represents TEM-20 but there is inconsistency and confusion between the full text and figure legend.
Fixed. We meant that there were two types of labeling the cells which did not affect the final concentration of the mutant strain.

Suggested alterations:
1. I strongly recommend an additional table specifying the genotypes of the different TEM strains and their MIC. It was difficult reading the paper without that information in a specific location/ having to look for that information embedded in the text or figure legends.*

As mentioned previously, we have decided to refer to the primary two strains in the paper as the "reference" strain and the "mutant" strain. We hope that this will help make the paper easier to read. In addition, we have added a table in the supplementary section describing all of the strains.

2. When first introducing β-lactams, it would be beneficial to clearly mention that they are bactericidal antibiotics as it allows for better understanding of later figures such as Figure S1. Otherwise, it could appear that concentration 2.2μg/mL is the lowest concentration that results in cell death because it shows a decrease in CFU.

Added the following sentence at line 46:

© European Molecular Biology Organization

β-lactams are bactericidal and therefore any bacteria that survive the treatment often go through the death phase.

3. I would recommend changing the colors of Figure S1 so that high drug concentrations are in red (cell death) and low drug concentrations are in green (cell growth) for a more intuitive understanding of the figure.
Fixed.

4. In the Sequencing summary table, it is mentioned that when evolving TEM-19 to 0.25μg/mL of drug, there is a "silent mutation at position 20 GCG -> GCT". I recommend being conservative and saying that this is an observed synonymous mutation unless it is definitely known that this mutation is also silent. Synonymous mutations can alter rate and efficiency of translation causing them to be non-silent.
Thank you! Fixed.

5. In Figure S2, is the color of the CFP histogram intended to be blue instead of green to specify that it is CFP counts and not YFP counts?

The figure is a print screen from the flow cytometer software, so changing the colors will require some time and we believe is not necessary.

6. In Figure S4, I highly recommend using the same scales for both the x and y-axis to provide a more honest representation of the data and allow for ease of comparison.

Thank you for this recommendation, but for the purpose of the uniform style across all figures we prefer to leave it as it is.

7. Why cite CLSI antimicrobial susceptibility testing for anaerobic bacteria when you are using E. coli, an aerobic bacterium?
Fixed.

Suggested Experiments: Nitrocefin Assay- β-lactamases can be secreted by bacteria into the media. As a control it would be good to test the amount of TEM enzyme in your media that could also be inactivating cefotaxime and altering the steady-state levels of this drug.

It has been shown before that β-lactamases are secreted by bacteria into the media (we added citation in the main text). We have also performed experiments (not published) confirming that β-lactamase is secreted into the media by filtering the spent media and performing either biological or chemical assays on the spent media without cells. For example, our experiments with fluorocillin (https://www.lifetechnologies.com/order/catalog/product/F33952) confirmed that the beta-lactamase is indeed released to the media and that the enzyme is degraded over time. However since these results have been published before and are not essential for the main points of this paper we prefer not to include those experiments here.

Reviewer #2:

The authors sought to understand the evolution of bacterial resistance through the analysis of a novel metric, termed here scMIC. In contrast to conventional knowledge typically associating bacterial population resistance with the MIC, or the minimum inhibitory concentration, the authors argued that the single cell MIC (scMIC) would serve as a better measure of evolutionary fitness. To demonstrate this point, the authors used plasmid-encoded Bla and determined population MIC or scMIC by varying the initial cell density, where low enough cell density would remove the cooperation ability for Bla to act as a public good. The authors found that the scMIC was always at least an order of magnitude smaller than the measured MIC both in liquid culture and solid phase.

The authors then demonstrated that selection within a bacterial population begins at the antibiotic concentration corresponding to the scMIC, not the MIC, which has particular relevance for the development of resistant populations in vivo. To investigate competition of a mixed bacterial

© European Molecular Biology Organization

population, the authors paired beta-lactamase plasmids and introduced various antibiotic concentrations below, at or above the scMICs of each harboring strain. They investigated the idea that antibiotic between the two scMIC values of competing strains will favor the one retaining the higher resistance, but once passed both scMIC levels, neither has a fitness advantage. Experimental results support this observation. To investigate this further, the authors implemented a C. elegans model to analyze population resistance in vivo, which was consistent with in vitro experiments as well. The authors then developed a simple mathematical model to explain the observations, which accurately predicts the relationship of MIC and cell density, as well as nonlinear relationship between scMIC and antibiotic. Lastly, the authors investigate the relationship between MIC and scMIC, demonstrating that the inoculum effect plays a crucial role in the discrepancy between scMIC and MIC. The authors conclude by stating the importance of understanding the evolution of bacterial antibiotic resistance, and how this metric allows for increased understanding of selection and advantage of populations under selective antibiotic pressure.

General Comments:
Although the idea of inoculum effect is well known, this work is novel in its attempt to derive a metric that is independent of the initial cell density. The key idea underlying the scMIC is that at a sufficiently low density, the cooperative nature of antibiotic degradation is negligible. If measured at such a density, the MIC reflects what is needed to kill individual cells. As a result, this scMIC is independent of cell density. The work is interesting and overall well presented. This study is timely, given the need for improved treatment strategies, insightful, given the need to better understand how population level dynamics affect drug responses, and well thought out. It merits publication with some clarifications and revisions.

We thank referee #2 for the careful reading of the manuscript and the overall encouraging assessment of the paper.

While I like the concept of scMIC, however, I have major reservations about the claim that it's the best metric to predict selection. For instance, the authors use this study to claim that scMIC is superior in order to quantify antibiotic resistance. Resistance can certainly be split in separate categories. However, population level resistance does exist, and for that MIC may still be relevant in order to understand larger-scale phenotypes, even if resistant 'evolution' begins at the scMIC. By the nature of the definition, scMIC seems an appropriate metric when there is negligible turnover of the antibiotic. In a mixture consisting of two populations with different scMICs, the longer-term dynamics of the two populations will be highly dependent on the subsequent turnover of the antibiotic by either or both populations. In this situation, the predictive power of scMIC is likely limited. It is unclear how the authors avoided this complication in their experiments (in vitro or in vivo). It would be helpful to clarify this point by additional modeling and/or experimental analysis, or better clarify the application context of scMIC.

To the point about population level resistance, the first comments to the previous reviewer apply. In short, we agree that population level resistance does exist, is relevant in many contexts, and that the MIC* is one way to quantify it.
To the point that the relevance of the scMIC is density-dependent, we believe that this is not true and mention it in the original manuscript (original line 208, new line 222: 'Our model agrees with the experimental finding that independent of initial cell density, selection favoring the competitor with the higher scMIC will begin when the antibiotic concentration approaches the scMIC of the less resistant strain (Fig. 6f).'). This is an important point, so we have stressed it throughout the manuscript.
In original line 228 (new line 243), after the words 'experiences cell death.', we have added the following sentences: " On the contrary, the antibiotic concentration at which selection starts does not depend strongly on the cell density. This makes sense since the periplasmic antibiotic concentration at the beginning of the experiment is independent of the cell density. The cell density does, however, alter the temporal dynamics of the antibiotic concentration over the course of the day, thus modifying the strength of selection favoring the strain with higher scMIC."
On the experimental side, in the new version of the manucript we stress that our competition experiments and evolution experiments are performed at cell densities that are closer to the MIC* cell density and not scMIC.

Finally, we would like to stress that in our experiments the antibiotic concentration is changing a great deal over the course of the bacterial culture death and growth. The scMIC is therefore powerful even when there is not "negligible turnover of the antibiotic".

Other specific comments:
1. All of the beta-lactamase used were TEM plasmid-born resistance. Are they under the same promoter?
Yes, the different versions of beta-lactamase in this study are all under the same promoter.
Are some constitutive and others inducible by the antibiotic? This could change interpretation of the results. I assume they were all constitutive; could the authors comment on how this would change with inducible resistance?

The promoter is expressed constitutively. If expression were induced by the antibiotic then there would be some lag of gene production due to the response time. By the time enough of the enzyme is created, some of the cells will be dead, but the overall advantage of higher resistant will be present assuming that the more resistant mutant is under the same inducible promoter as a reference strain. While the interaction of gene induction dynamics with the antibiotic break down dynamics may be interesting, we would prefer not to discuss it in this paper as it is tangential to the points we are trying to make.
Also, what does the scMIC look like for a sensitive strain?
The scMIC and MIC* are very similar for a sensitive strain (see Figure 7).

2. Although MIC is a potent indicator, beta-lactams actually respond more to time above MIC than concentration above MIC. Do the authors have any indication whether the time spent above the scMIC has the same effect? This is especially relevant for beta-lactams and would be interesting to see.

We agree with the reviewer that time above the MIC is thought to be a clinically important quantity because maximizing this time leads to the most effective elimination of the bacterial population. However, given the collective mode of enzymatic breakdown considered in this study, we believe that it is really the time spent above the scMIC that drives both the population and evolution dynamics. Indeed, the difference between this time for two different strains with different scMIC values is precisely what drives the competition experiments that we have performed.

3. While I understand the rationale of the term single-cell MIC, it is still empirically determined with a small bacterial population (~500cells/mL). I would prefer the authors use a different term for consistency.

In the original version of the manuscript we demonstrated that at antibiotic concentrations equal to the scMIC is when colonies no longer form on an agar plate. While we believe that this is sufficient to demonstrate that the scMIC is indeed a property of single cells, in the new version of the manuscript we have performed microscopy (figure 2) to demonstrate directly that this is the case.

4. Although interesting, the density will never be so low in vivo and the MIC is still more relevant for clinical applications. Can the authors comment on how this might affect clinical interpretations of MIC? In particular, how would one go about using scMIC to guide clinical intervention? Some comments on this point would be useful.

In the new version of the manuscript we tried to make it clearer that while scMIC is measured at very low cell densities, its evolutionary implications are true at all cell densities (as illustrated in Fig. 3 and Fig. 4 and Fig. 6). For clinical intervention, in the short term, evolutionary considerations may not be dominant, and instead a clinician needs to know how much of the antibiotic should be given to the patient (which may involve population level resistance). However, we believe that on the policy scale and in agriculture the conclusions of our study might be useful.

5. An increase in scMIC vs an increase in MIC may not be a fair comparison. If one does not occur simultaneously with the other, then different mechanisms are at play and both metrics would provide valuable insight into population level resistance. As noted above, in a longer term, as the bacterial

populations degrade the antibiotic (which underlies the inoculum effect), the predictive power of the scMIC for the ultimate evolution dynamics may be limited.

We agree with the point that the more information that is collected, the more we can say about the resistance. However, in this paper we are trying to focus and concentrate on the evolutionary properties of these metrics. Our claim is that scMIC is superior to MIC* for predicting evolution at any cell densities. We acknowledge that antibiotic resistance can be viewed from many different perspectives, including population-level resistance, for which MIC* might be more useful than scMIC. The antibiotic is degraded over time in our experiments, and this is taken into account in our model.

6. When the authors state "selection for increase scMIC", what's the mechanism for the increased scMIC? It would be useful to provide some experimental evidence (e.g. mutations underlying this increase). As noted above, selection for MIC and scMIC may occur at different levels. I presume scMIC is happening at the genetic level, meaning that population level conclusions may not be fair to draw.

In the competition experiments of known strains, the mechanism for increased scMIC or MIC* is genetic since we know what the strains are. In the evolutionary experiments we have identified de novo mutations within the beta-lactamase gene that have fixed and are likely the cause of the increase in scMIC.

Reviewer #3:

The authors of this manuscript introduce the concept of single cell MIC (scMIC), which is the MIC value measured at low (~ 500 cell/ml) cell densities. They show that in the case of the β-lactam antibiotic cefotaxime, which is inoculum size dependent, the scMIC is at least one order of magnitude lower than the standard MIC measured at thousand-fold higher cell densities. They also prove that at low cell densities the MIC does not depend on the cell concentration any more, providing a more robust data. This is due to the fact that at low cell densities the cooperative behavior of the cells cannot mask the real resistance level (scMIC) of the individual cells. Therefore, the authors suggest that scMIC is a superior metric for quantifying resistance, compared to the standard MIC. They also claim that at antibiotic concentrations lower than the scMIC the applied antibiotic does not affect the fitness of the cells, and therefore selection for resistant mutants starts at concentrations close to or above the scMIC. To support this hypothesis adaptive laboratory evolution experiments were carried out in the presence of cefotaxine, as well as competition experiments with β-lactam resistant E. coli strains with different resistance levels. The authors claim that based on the results from these experiments the scMIC "predicts the direction of selection and also specifies the antibiotic concentration at which selection begins to favor new mutants"

Major concerns:
1) Previous studies (Gullberg et al., 2011, 2014; Hughes and Andersson, 2012) have shown that selection of resistant bacteria can occur at antibiotic concentrations several hundred-fold below the MIC. Specifically, Gullberg and his colleagues (Gullberg et al., 2014) have shown that antibiotic concentrations much below the MIC are sufficiently high for maintaining multidrug resistant, extended-spectrum β-lactamase (ESBL) containing plasmids. They proved this for antibiotics with different mechanism of action, including those where the inoculum size dependence of the MIC is absent. In the latter case the standard MIC and the scMIC are the same.
*The authors of the present manuscript themselves state that the standard MIC "may not perform well as a measure of evolutionary fitness, even where resistance is not density-dependent. For example, a recent study demonstrated that sub-MIC*levels of tetracycline, aminoglycosides, and fluoroquinolone antibiotics can select for cells carrying an antibiotic resistance plasmid". With this statement they contradict themselves because in the case of antibiotics with no inoculum size effect the scMIC and MIC do not differ from each other, which means that selection occurs below the level of scMIC.*
So my major concern regards the basic prediction of the authors that at concentrations below the scMIC antibiotics do not affect the fitness of the cells, and consequently selection does not act at concentrations below the scMIC. However, the authors themselves claim in line 211 that there is a fitness effect of the antibiotic below the scMIC concentration.

We agree that our discussion of these effects was not sufficiently clear. We are arguing that there are two distinct mechanisms that lead to selection for antibiotic resistance at low (sub-MIC*) antibiotic concentrations: 1) collective inactivation and 2) gradual decrease in growth with antibiotic concentration (as compared to a sharp hill function that looks like a "step"). Importantly, it is possible that these two mechanisms could both be present, although in the cases studied in our paper we believe that the first mechanism is dominant.
To make this point more clear, we have modified the following paragraph between 303 and 304 lines of the original manuscript (new lines 333-341):
In this paper, we have assumed that the growth rate falls as a step-function with increasing antibiotic concentrations, which is a reasonable approximation for most β-lactams and for a variety of other antibiotics(Wiuff *et al*, 2005) (Johnson & Levin, 2013). However, for some antibiotics (such as tetracycline), the growth rate falls gradually with increasing antibiotic concentrations. In particular, very low concentrations of antibiotic have a modest but potentially significant effect on bacterial growth. In this situation, it is possible to get selection for antibiotic resistance at sub-MIC concentrations of antibiotic, even in the absence of collective inactivation of the antibiotic (in which case the scMIC is equal to the traditional MIC). For example, a recent study demonstrated that sub-MIC levels of tetracycline, aminoglycosides, and fluoroquinolone antibiotics can select for cells carrying an antibiotic resistance plasmid(Gullberg *et al*, 2011). The resistance mechanism in this previous study was not cooperative, and inoculum effects were not observed; selection occurred when growth inhibition of sensitive strains at sub-MIC* antibiotic concentrations was greater than the growth disadvantage associated with carrying the plasmid conferring resistance, a point designated by the authors as the minimal selective concentration (MSC). Collective antibiotic degradation is therefore not the only mechanism for sub-MIC selection for antibiotic resistance. It is worth noting, however, that Gullberg et al were studying antibiotic concentrations required to select for resistance relative to a sensitive population, whereas in the current study we have been exploring selection for increased resistance in a population that already has some level of resistance.

2) How much the usability of the scMIC as a metric for quantifying resistance evolution can be extrapolated from cefotaxime to other antibiotics with no inoculum size effect? I think this cannot be done, because the distinction between scMIC and MIC is relevant only for resistance mechanisms governed by enzymatic inactivation of the drug. Even in the case of cefotaxime, when the resistance level provided by a β-lactamase variant was low, the scMIC and MIC values were nearly the same. In my opinion the results presented are not systematic enough, and in the above pointed aspects are contradictory as well.

Indeed if there is no inoculum size effect then the scMIC and MIC* are the same by definition (assuming sharp Hill function for growth rate as a function of antibiotic concentration). In this case the MIC* would work to predict the direction of selection, but we would claim that in this case it works because it is the same as the scMIC.

2nd Editorial Decision 11 May 2015

Thank you again for submitting your work to Molecular Systems Biology. We have now heard back from the two referees who agreed to evaluate your manuscript. As you will see below, while reviewer #2 thinks that the revised manuscript is significantly improved, reviewer #3 still has some remaining concerns regarding the prediction of selection by the scMIC. We have circulated the reports to all reviewers as part of our 'pre-decision cross-commenting' policy. During this process, reviewer #2, mentioned: "As indicated in my comments, I do believe the authors should further clarify the context in which the scMIC can serve as a predictive metric for the direction of selection. Based on my reading of the manuscript, it appears that the metric is a good predictor if antibiotic concentrations beyond the range of two scMIC values (of the competing strains) affect the two strains in the same manner (or very similar manner). In their model, they made this simplifying assumption. However, if an antibiotic concentration lower than both scMIC values causes differential growth inhibition, it would still cause selection. That may account for the observations mentioned by Reviewer #3. That said, overall I find the paper significantly distinct from the literature (including those mentioned by the reviewer #3). It provides a mechanistic basis on how antibiotic at concentrations near scMIC contribute to selection of more resistant strains. In terms of the depth of the study, I find the paper quite substantial. Even though testing on additional

antibiotics would be useful, I don't think it should be a critical requirement for the paper to be published." Moreover, reviewer #2 pointed out that, in line with the comments in his/her report, it is important to clarify the predictive power of the scMIC in light of the literature. As such, we think that further experiments are not required (unless they are already available, in which case they can of course be included), but we would ask you to include further discussion and clarifications regarding the points raised by the two referees.

Reviewer #2:

I remain enthusiastic about the study, which I find exciting and significant. Overall, the responses and revision by the authors have clarified some of the confusions I had and cleared some reservations, mostly on the technical side. I would recommend the paper to be published though I still have some minor suggestions for the authors, mostly on the presentation of arguments.

1. The scMIC metric
A major contribution is the derivation of scMIC and experimental demonstration of this metric. In contrast to MIC, which is typically determined empirically, the scMIC is uniquely determined by several fundamental parameters (efficiency of Bla and permeability of cell membrane) that are independent of the cell density. In particular, the aspect I like most about the paper is the two simple relationships they derived:
One between the scMIC and the parameters characterizing the efficiency of Bla.
One between the scMIC and the MIC at a particular cell density. I suggest the authors also to include the latter in the main text.
These two relationships would allow the estimation of both scMIC and MIC if the fundamental parameters and the cell density are known.

The authors provided further evidence on the notion of single-cell MIC (scMIC). In the original MS, it was measured based on the plateauing of MIC as the population size shrinks (to ~500 cells/ml). The previous measurements were convincing but not directly done at the single-cell level, thus my reservation on the term "scMIC". With the new evidence, I'm comfortable with the term.

2. Predicting of direction of selection by scMIC
They also clarified on how scMIC represents a better metric than MIC for predicting direction of selection. This conclusion is overall convincing to me. In their writing, however, I would recommend the authors to be more precise in their language to avoid potential strawman arguments. In this regard, I share some of the reservations raised by Reviewer 1 and I feel the authors could further tune down on their claims.

Indeed, both their modeling and experimental results suggest that selection starts to act around the scMIC. The intuition underlying this, however, is somewhat blurry. The key mechanistic basis for this idea is that, for a particular strain, scMIC represents the concentration when the net growth rate of a population turns negative (page 3 of their Supp Materials). Beyond this, there appears to be another key assumption: the growth rate is the same positive constant when [antibiotic] < scMIC; likewise, the death rate is also the same constant when [antibiotic] > scMIC. If the antibiotic concentration lands between the scMIC values of two strains, it's clear that selection would favor the one with higher scMIC. However, if the [antibiotic] is slightly higher than both (or lower than both), the outcome of selection would depend on how the antibiotic specifically change the growth rates or the death rates. They alluded to this point between lines 226 and 228. Regardless, the authors should consider further clarifying the context where scMIC predicts direction of selection.

Other minor points:
1. In quite a few panels, they used gray bars to indicate the values of scMIC. These are of varying width -- does the width of a gray bar mean anything?
2. Line 37. As noted above, I don't think it is generally thought that the MIC is the minimum inhibitory concentration at which selection begins, as there are tons of studies showing sub-inhibitory concentrations of antibiotics have counter intuitive effects.
3. Line 48: typo, they cited Yurtsev 2013 twice
4. Generally, their conclusions are applicable to beta lactams antibiotics. But claiming scMIC is

© European Molecular Biology Organization

robust for predicting selection dynamics seems too broad a conclusion to draw without further evidence. They don't need to make such claims for the paper to be significant.
5. In describing inoculum effect, they authors might note a recent review paper on this topic: Meredith et al, Nat Chem Biol 2015, Collective antibiotic tolerance: mechanisms, dynamics and intervention

Reviewer #3:

The revised version is disappointing: authors did not introduce any conceptual change related to the problems raised. I have to reiterate my concerns:
1) The main conclusion of the manuscript, i.e. that under scMIC antibiotic concentration selection does not favor the emergence of resistant mutants stands against the results of more thorough and systematic previous works (Gullberg et al., 2011, 2014; Hughes and Andersson, 2012), where the MIC* of the used antibiotics equals to the scMIC.
2) The main message of the manuscript, that "The scMIC accurately predicts the evolutionary behavior of bacterial populations exposed to an antibiotic" is dubious to say the least. The scMIC (a more accurate measure of the MIC) has no predictive power on the evolution of antibiotic resistance.

3) The results of the experiments intending to prove that there is no selection under scMIC antibiotic concentration are unsatisfactory, for two reasons:
a) The time period of the competition experiments was too short (24hrs) to assess small fitness differences that could be evolutionarily relevant. Small fitness differences (e.g. on the order 0.01-0.001 cannot be distinguished from noise). In the previously cited work (Gullberg et al., 2011) competition experiments were carried out for up to 40 generations to show fitness differences at subMIC concentrations.
b) Figure 4b indicates that there is already a two fold MIC change even when adaptive laboratory evolution experiment was carried out below scMIC concentration, which contradicts with the main conclusion of the paper. It is not clear whether MIC would further increase if the evolutionary experiment were carried out longer.
4) Even if the presented results were satisfactory, I would sustain my opinion that the study is not systematic enough, as only a single antibiotic was involved.

2nd Revision - authors' response 16 June 2015

Below we address the remaining concerns expressed by the referees and describe how we have modified the text in response.
Thank you.

Reviewer #2:

I remain enthusiastic about the study, which I find exciting and significant. Overall, the responses and revision by the authors have clarified some of the confusions I had and cleared some reservations, mostly on the technical side. I would recommend the paper to be published though I still have some minor suggestions for the authors, mostly on the presentation of arguments.

We are glad that reviewer #2 is enthusiastic about our study and feels that most of the concerns have been addressed.

1. The scMIC metric
A major contribution is the derivation of scMIC and experimental demonstration of this metric. In contrast to MIC, which is typically determined empirically, the scMIC is uniquely determined by several fundamental parameters (efficiency of Bla and permeability of cell membrane) that are independent of the cell density. In particular, the aspect I like most about the paper is the two simple relationships they derived:
One between the scMIC and the parameters characterizing the efficiency of Bla.
One between the scMIC and the MIC at a particular cell density. I suggest the authors also to

include the latter in the main text.
These two relationships would allow the estimation of both scMIC and MIC if the fundamental parameters and the cell density are known.
We agree that these relationships can be insightful. However, at high antibiotic concentrations and therefore high cell densities of the inoculum effect curve, many other mechanisms, not included in our model might be at play and therefore the relationship might not be that useful and therefore probably does not deserve to be in the main text. Nevertheless, we expanded the discussion of the shape of the inoculum effect in the main text.
We added the following paragraph after line 221 of the original manuscript:
"In our model, the inoculum effect curve can be derived analytically in the limit of low and high cell densities. These two regimes are determined by how MIC compares to K_M. If MIC is smaller than K_M then the hydrolysis rate increases proportionally to the antibiotic concentration, and as a result the measured MIC is an exponential function of the initial cell density:

$$MIC = scMIC\ exp(\frac{n_0 V_{max}}{K_M}\frac{\gamma t_{20}}{\gamma + \gamma_d})$$

Where the n_0 is the initial cell density, γ is the growth rate, γ_d is the death rate, and t_{20} is the time before evaluation of the MIC (usually 20 hours). At higher antibiotic concentrations, the hydrolysis rate becomes independent of the antibiotic concentration, and the model that MIC increases linearly with the initial cell density. However, the experimentally measured MIC grows slower than linearly with the initial cell density. This phenomenon could be explained by the fact that in this regime the population spends a significant amount of time in the death phase and degradation of the released enzyme could become significant (Supp)."

The authors provided further evidence on the notion of single-cell MIC (scMIC). In the original MS, it was measured based on the plateauing of MIC as the population size shrinks (to ~500 cells/ml). The previous measurements were convincing but not directly done at the single-cell level, thus my reservation on the term "scMIC". With the new evidence, I'm comfortable with the term.

We are glad that our new experimental data has convinced reviewer #2 that our use of the phase single-cell MIC is appropriate.

2. Predicting of direction of selection by scMIC
They also clarified on how scMIC represents a better metric than MIC for predicting direction of selection. This conclusion is overall convincing to me. In their writing, however, I would recommend the authors to be more precise in their language to avoid potential strawman arguments. In this regard, I share some of the reservations raised by Reviewer 1 and I feel the authors could further tune down on their claims.

Indeed, both their modeling and experimental results suggest that selection starts to act around the scMIC. The intuition underlying this, however, is somewhat blurry. The key mechanistic basis for this idea is that, for a particular strain, scMIC represents the concentration when the net growth rate of a population turns negative (page 3 of their Supp Materials). Beyond this, there appears to be another key assumption: the growth rate is the same positive constant when [antibiotic] < scMIC; likewise, the death rate is also the same constant when [antibiotic] > scMIC. If the antibiotic concentration lands between the scMIC values of two strains, it's clear that selection would favor the one with higher scMIC. However, if the [antibiotic] is slightly higher than both (or lower than both), the outcome of selection would depend on how the antibiotic specifically change the growth rates or the death rates. They alluded to this point between lines 226 and 228. Regardless, the authors
should consider further clarifying the context where scMIC predicts direction of selection.

We apologize that we have not been sufficiently explicit / clear about our assumptions. The explanation supplied by reviewer #2 above is indeed accurate. To address this we have made changes after line 124 in the original manuscript and in the paragraph starting at line 321 of the original manuscript. To see the discussion of these points, see our answers to Reviewer #3 below.

Other minor points:

1. In quite a few panels, they used gray bars to indicate the values of scMIC. These are of varying width -- does the width of a gray bar mean anything?
The width corresponds to our confidence in the number (standard error of the mean). The widths vary because different strains have different errors and also because some of the plots have different range of the axis.

2. Line 37. As noted above, I don't think it is generally thought that the MIC is the minimum inhibitory concentration at which selection begins, as there are tons of studies showing sub-inhibitory concentrations of antibiotics have counter intuitive effects.
We agree. We will softer to “sometimes thought” in line 37.

3. Line 48: typo, they cited Yurtsev 2013 twice
Thank you! Fixed.

4. Generally, their conclusions are applicable to beta lactams antibiotics. But claiming scMIC is robust for predicting selection dynamics seems too broad a conclusion to draw without further evidence. They don't need to make such claims for the paper to be significant.
After further consideration of this we agree that we should soften the speculation on this point. In particular, at line 53 of the original manuscript we added “at least in β-lactams”, at line 299 – “for β-lactams”.

5. In describing inoculum effect, they authors might note a recent review paper on this topic: Meredith et al, Nat Chem Biol 2015, Collective antibiotic tolerance: mechanisms, dynamics and intervention

Added.

Reviewer #3:

The revised version is disappointing: authors did not introduce any conceptual change related to the problems raised. I have to reiterate my concerns:
1) The main conclusion of the manuscript, i.e. that under scMIC antibiotic concentration selection does not favor the emergence of resistant mutants stands against the results of more thorough and systematic previous works (Gullberg et al., 2011, 2014; Hughes and Andersson, 2012), where the MIC of the used antibiotics equals to the scMIC.*
Reviewer #2 has highlighted the assumption that we are making (that the growth rates of the mutants are equal below the scMIC). This assumption seems to apply to our strains, but it may break down in other contexts. It is also important that in our experiments we are considering point-mutations in an already existing protein. In this case there is little to no “cost” associated with the mutation conferring higher level resistance. In the experiments referenced above the authors were considering antibiotics that produce gradual decrease in growth, where the selection for increase of resistance starts at very low antibiotic concentrations. To clarify this further, we modify main text by expanding the sentence on lines 196-199 of the original manuscript to a paragraph:
“We experimentally found that for our TEM strains in cefotaxime, this growth rate function can be approximated as a step function: cells divide at a normal rate until the antibiotic concentration in the periplasmic space is above some value a_{crit}, at which point cells die at a rate ~ $2hr^{-1}$ (Fig. 6c,6d, S1,S5).
Increased resistance in our experiments is conferred with no cost because it is acquired by one or a few point mutations in β-lactamase. The benefit of the higher resistance (and therefore lower periplasmic concentrations for a given concentration outside the cell) is realized only when the antibiotic concentration is high enough so that the reference strain dies and low enough so that the mutant strain still grows. Outside of this antibiotic concentration range, there is no benefit of higher resistance.”

We also rewrote and reorganized most parts of discussion to make the assumptions that are crucial to the conclusions clear.

2) The main message of the manuscript, that "The scMIC accurately predicts the evolutionary

© European Molecular Biology Organization

behavior of bacterial populations exposed to an antibiotic" is dubious to say the least. The scMIC (a more accurate measure of the MIC) has no predictive power on the evolution of antibiotic resistance.
We disagree. First, the scMIC is not merely a more accurate measure of the MIC, as the group-based resistance that is quantified by the MIC may still be relevant for many purposes. Second, we believe that our experiments demonstrate 1) that selection for new mutants begins at antibiotic concentrations around the scMIC and 2) that in this case it is mutants that increase the scMIC that will spread (even if the MIC* decreases). In our opinion, these results demonstrate "predictive power".
We tried to clarify this point by explicitly narrowing down our definition of fitness. In the line 29 of the new manuscript, we added the following sentence:
"Since the term "fitness" can have different meanings depending on the context, we clarify that here fitness refers to the ability of a strain to spread in the presence of an antibiotic."

3) The results of the experiments intending to prove that there is no selection under scMIC antibiotic concentration are unsatisfactory, for two reasons:
a) The time period of the competition experiments was too short (24hrs) to assess small fitness differences that could be evolutionarily relevant. Small fitness differences (e.g. on the order 0.01-0.001 cannot be distinguished from noise). In the previously cited work (Gullberg et al., 2011) competition experiments were carried out for up to 40 generations to show fitness differences at subMIC concentrations.
We agree that 1 day long experiments cannot measure very small fitness differences. However, we are looking at different effect and our experiments are good enough for this purpose. While there could be small fitness differences at lower antibiotic concentrations, there is no theoretical reason to believe in it (we are mostly interested in single amino acid substitutions). And even if this if happening, it does not deny the fact that much stronger selection is happening on sorter time scales due to the reasons we describe in the manuscript.

b) Figure 4b indicates that there is already a two fold MIC change even when adaptive laboratory evolution experiment was carried out below scMIC concentration, which contradicts with the main conclusion of the paper. It is not clear whether MIC would further increase if the evolutionary experiment were carried out longer.
We agree that if the evolutionary experiment was the only experimental support of our result, the existing data will be a very week argument towards our point. However, we present a variety of techniques to demonstrate the main message of our study and collectively, we believe, they are convincing.

4) Even if the presented results were satisfactory, I would sustain my opinion that the study is not systematic enough, as only a single antibiotic was involved.
We agree that it will be valuable to extend our results to other antibiotics, but we believe that the insight that we have obtained into the evolution of resistance against cefotaxime will be of interest to a broad audience as similar effects may be observed for the evolution of other proteins that breaks down antibiotic.

© European Molecular Biology Organization
